# Leveraging AI-Driven Neuroimaging Biomarkers for Early Detection and Social Function Prediction in Autism Spectrum Disorders: A Systematic Review

**DOI:** 10.3390/healthcare13151776

**Published:** 2025-07-22

**Authors:** Evgenia Gkintoni, Maria Panagioti, Stephanos P. Vassilopoulos, Georgios Nikolaou, Basilis Boutsinas, Apostolos Vantarakis

**Affiliations:** 1 Department of Educational Sciences and Social Work, University of Patras, 26504 Patras, Greece; stephanosv@upatras.gr (S.P.V.); gnikolaou@upatras.gr (G.N.); 2 Division of Population Health, Health Services Research & Primary Care (LS), University of Manchester, Manchester M13 9PL, UK; maria.panagioti@manchester.ac.uk; 3 Department of Business Administration, University of Patras, 26504 Patras, Greece; vutsinas@upatras.gr; 4 Department of Medicine, University of Patras, 26504 Patras, Greece; avanta@upatras.gr

**Keywords:** autism spectrum disorder, artificial intelligence, neuroimaging, biomarkers, electroencephalography, functional connectivity, early detection, social function, developmental trajectories

## Abstract

**Background**: This systematic review examines artificial intelligence (AI) applications in neuroimaging for autism spectrum disorder (ASD), addressing six research questions regarding biomarker optimization, modality integration, social function prediction, developmental trajectories, clinical translation challenges, and multimodal data enhancement for earlier detection and improved outcomes. **Methods**: Following PRISMA guidelines, we conducted a comprehensive literature search across 8 databases, yielding 146 studies from an initial 1872 records. These studies were systematically analyzed to address key questions regarding AI neuroimaging approaches in ASD detection and prognosis. **Results**: Neuroimaging combined with AI algorithms demonstrated significant potential for early ASD detection, with electroencephalography (EEG) showing promise. Machine learning classifiers achieved high diagnostic accuracy (85–99%) using features derived from neural oscillatory patterns, connectivity measures, and signal complexity metrics. Studies of infant populations have identified the 9–12-month developmental window as critical for biomarker detection and the onset of behavioral symptoms. Multimodal approaches that integrate various imaging techniques have substantially enhanced predictive capabilities, while longitudinal analyses have shown potential for tracking developmental trajectories and treatment responses. **Conclusions**: AI-driven neuroimaging biomarkers represent a promising frontier in ASD research, potentially enabling the detection of symptoms before they manifest behaviorally and providing objective measures of intervention efficacy. While technical and methodological challenges remain, advancements in standardization, diverse sampling, and clinical validation could facilitate the translation of findings into practice, ultimately supporting earlier intervention during critical developmental periods and improving outcomes for individuals with ASD. Future research should prioritize large-scale validation studies and standardized protocols to realize the full potential of precision medicine in ASD.

## 1. Introduction

Autism spectrum disorder (ASD) is a complex neurodevelopmental condition characterized by persistent challenges in social communication and interaction alongside restricted and repetitive patterns of behavior, interests, or activities [1,2,3]. Families caring for individuals diagnosed with ASD serve as the primary and ongoing sources of support across their lifespan, facing significantly greater demands compared to those caring for typically developing children. Early detection and intervention are crucial for improving long-term outcomes. However, current diagnostic procedures often rely heavily on behavioral observations that may not manifest until toddlerhood or beyond, delaying crucial early interventions [4,5,6].

ASD is clinically diagnosed and defined by predetermined criteria stipulated in the *Diagnostic and Statistical Manual of Mental Disorders*, Fifth Edition, Text Revision (DSM-5-TR) and International Classification of Diseases, Eleventh Revision (ICD-11), which stress persistent deficits in social communication and social interaction across many contexts and restricted, repetitive patterns of behavior, interests, or activities. Severity is assessed by using specifiers for the degree of support required: requiring support, requiring substantial support, or requiring very substantial support, based on social communication impairment and restricted repetitive behaviors. Differential diagnosis requires consideration of other neurodevelopmental disorders, such as intellectual disability, global developmental delay, attention-deficit/hyperactivity disorder, and specific language impairment, as well as medical conditions like Rett syndrome and fragile X syndrome. Standardized instruments such as the Autism Diagnostic Observation Schedule-2 (ADOS-2), Autism Diagnostic Interview—Revised (ADI-R), and Childhood Autism Rating Scale (CARS) form part of current diagnostic assessments, supplemented by thorough developmental and functional evaluations. Associated assessments commonly include genetic testing to determine underlying syndromes, speech-language pathology evaluation, occupational therapy evaluation, and cognitive testing to define the individual’s entire neurodevelopmental profile and guide intervention planning [7,8].

The advent of advanced neuroimaging techniques combined with artificial intelligence (AI) methodologies has opened promising avenues for identifying objective biomarkers that could potentially detect ASD before behavioral symptoms fully manifest [9,10,11,12,13]. Neuroimaging studies have consistently revealed structural and functional brain differences in individuals with ASD, suggesting the potential for developing reliable biomarkers. A biomarker is defined as a measurable biological characteristic that serves as an indicator of normal biological processes, pathogenic processes, or pharmacologic responses to therapeutic interventions. In the context of neuroimaging and ASD research, biomarkers refer to objective, quantifiable neural measurements derived from brain imaging data that can reliably distinguish individuals with ASD from typically developing individuals, predict developmental outcomes, monitor treatment responses, or identify individuals at risk for developing ASD before clinical symptoms fully manifest. However, the heterogeneity of ASD presentations and the complexity of neuroimaging data present significant challenges in translating research findings into clinically applicable diagnostic and prognostic tools [14,15,16,17].

Recent technological advancements in artificial intelligence, encompassing traditional machine learning, deep learning, graph neural networks, ensemble methods, transfer learning, and explainable AI approaches, have remarkably enhanced our ability to analyze complex neuroimaging data [18,19,20,21]. These diverse computational methodologies include support vector machines, convolutional and recurrent neural networks, attention mechanisms, multimodal fusion techniques, federated learning frameworks, and Bayesian approaches, each offering unique advantages for different aspects of neuroimaging analysis. These computational approaches can identify subtle patterns and relationships within multimodal neuroimaging data that may not be apparent through conventional analysis methods. By leveraging AI-driven analysis of neuroimaging data, researchers aim to develop objective biomarkers that could facilitate earlier diagnosis, more accurate prognosis, and personalized intervention planning for individuals with ASD [22,23,24,25].

The potential impact of combining neuroimaging and AI extends beyond early detection, including the prediction of social functioning outcomes, which represents a core challenge in ASD. Social functioning difficulties significantly impact the quality of life and long-term consequences for individuals with ASD, yet current methods for predicting developmental trajectories remain limited [26,27]. AI-driven neuroimaging biomarkers could potentially identify neural signatures associated with specific dimensions of social functioning, enabling more targeted and effective interventions [28,29,30].

Moreover, patient and family caregivers often express pressing requirements for support and information regarding prognosis and intervention planning during clinical visits. Integrating neuroimaging biomarkers into clinical practice could provide valuable objective information to complement traditional assessments, potentially reducing diagnostic uncertainty and facilitating more personalized care recommendations. Minimizing public health-related stigma around neurodevelopmental disorders like ASD is a fundamental prerequisite for ensuring that affected individuals and their families seek and receive appropriate support [31,32,33,34].

Harnessing technology’s capacity to establish and validate biomarkers can facilitate more objective, precise, and earlier detection of ASD. The integration of established neuroimaging technologies with evolving AI algorithms and enhanced computational capabilities represents a valuable expansion of our toolkit for understanding and addressing ASD. These approaches broadly refer to using data, information, and computational technologies to enhance diagnostic accuracy, improve prognostic capabilities, and better support individuals with ASD and their families [35,36,37].

After the recent surge in research exploring the intersection of AI and neuroimaging in ASD, there has been a burgeoning effort to develop novel technological solutions to resolve the issues arising from delayed diagnosis and limited prognostic information. Due to the heterogeneity of ASD presentations and the complexity of brain development, researchers are increasingly adopting multimodal approaches that integrate data from multiple sources to develop more robust and sensitive biomarkers. Therefore, AI-driven neuroimaging approaches may represent critical tools for researchers, clinicians, and, ultimately, individuals with ASD and their families [38,39,40].

This systematic review aims to comprehensively synthesize the current research on AI-driven neuroimaging biomarkers for the early detection and prediction of social function in ASD. By examining methodological approaches, evaluating the strength of evidence, and identifying promising directions for future research, this review seeks to provide an evidence-based perspective on the potential of neuroimaging biomarkers to transform our approach to ASD diagnosis and intervention. We also aim to identify the most promising neuroimaging modalities, AI methodologies, and biomarker candidates for advancing the field toward clinical application.

## 2. Literature Review

### 2.1. Understanding Autism Spectrum Disorder and Neuroimaging Approaches

ASD is a neurodevelopmental condition characterized by persistent deficits in social communication and interaction combined with restricted, repetitive behavior patterns, interests, or activities. Current prevalence estimates indicate that approximately 1 in 54 children in the United States has been identified with ASD, with a male-to-female ratio of 4:1. The neurobiological basis of ASD involves complex genetic architecture, with over 100 genes implicated, along with epigenetic mechanisms and environmental factors contributing to its heterogeneous presentation. Neuroimaging technologies have significantly advanced our understanding of the neurobiological underpinnings of ASD [41,42,43,44,45]. Multiple imaging modalities have been employed to characterize the structural and functional brain alterations associated with the disorder:

Structural MRI (sMRI) studies have identified macroscopic neuroanatomical differences in ASD, including regional volumetric abnormalities, cortical thickness variations, and atypical gyrification patterns. Meta-analyses have consistently demonstrated altered neurodevelopmental trajectories characterized by early brain overgrowth, followed by normalization or deceleration in adolescence. Region-specific alterations have been observed in the frontal and temporal cortices, the amygdala, the cerebellum, and the corpus callosum, particularly in brain regions involved in social cognition and language processing [46,47,48,49,50,51].

Diffusion tensor imaging (DTI) has revealed white matter microstructural abnormalities in ASD, with reduced fractional anisotropy (FA) and increased mean diffusivity (MD) in multiple white matter tracts. These alterations are particularly pronounced in pathways connecting social brain regions, including the superior longitudinal fasciculus, inferior frontal–occipital fasciculus, and corpus callosum. Tractography analyses have demonstrated reduced structural connectivity between the frontal, temporal, and parietal regions involved in social information processing [52,53,54,55,56,57,58,59].

Functional MRI (fMRI) studies have identified altered neural activation patterns during social cognition tasks, including face processing, theory of mind, and joint attention. Task-based fMRI has revealed hypoactivation in brain regions comprising the “social brain network,” including the superior temporal sulcus (STS), the fusiform gyrus, the amygdala, and the medial prefrontal cortex (mPFC). Resting-state fMRI has demonstrated atypical functional connectivity patterns, including reduced long-range and increased local connectivity, potentially reflecting altered neural information integration [60,61,62,63,64,65,66].

Electroencephalography (EEG) has documented atypical neural oscillations and event-related potentials (ERPs) in ASD. Specific ERP components, such as P300, N170, and mismatch negativity (MMN), show altered amplitude and/or latency in response to social stimuli. Spectral analyses have revealed abnormalities in gamma-band activity, potentially reflecting imbalances in excitatory–inhibitory neurotransmission. Functional connectivity analyses using EEG have identified reduced long-range synchronization and increased local synchronization, findings consistent with those from fMRI [67,68,69,70,71,72].

Magnetoencephalography (MEG) studies have provided temporally precise characterization of neural processing abnormalities in ASD, focusing on the early stages of sensory processing. Documented alterations in M100 responses to auditory stimuli and atypical visual evoked field (VEF) responses to face stimuli suggest fundamental differences in the speed and efficiency of neural information processing [73,74,75,76,77,78,79].

These neuroimaging findings collectively suggest that ASD involves complex network-level disruptions rather than focal abnormalities in discrete brain regions. The developmental timing and specific patterns of these neurobiological alterations may significantly influence symptom expression and severity, highlighting the potential value of neuroimaging biomarkers for early detection and outcome prediction [80,81,82,83,84,85].

### 2.2. Social Function Deficits in Autism Spectrum Disorder

Social function deficits constitute a core diagnostic feature of ASD and significantly impact long-term outcomes across educational, occupational, and interpersonal domains. These deficits manifest across multiple dimensions of social cognition and behavior:

Social–emotional reciprocity deficits involve impairments in initiating and maintaining reciprocal social interactions, sharing emotions, and engaging in conversational turn-taking. Quantitative assessments using standardized instruments, such as the Social Responsiveness Scale (SRS) and the Autism Diagnostic Observation Schedule (ADOS), have consistently documented significant impairments in these domains, which are correlated with functional outcomes [86,87,88,89,90,91,92].

Nonverbal communicative behaviors used for social interaction are frequently impaired in ASD, including atypical eye contact, facial expressions, body posture, and gestures. Eye-tracking studies have documented a reduction in attention to social stimuli, particularly faces and eyes, with a corresponding preference for non-social aspects of visual scenes. Motion capture analyses have identified subtle abnormalities in the production and perception of social movements, including atypical kinematics of gestures and facial expressions [93,94,95,96,97,98].

Social cognition deficits include impairments in theory of mind (ToM), emotion recognition, and social perception. Functional neuroimaging studies during ToM tasks have revealed hypoactivation in the mPFC, temporoparietal junction (TPJ), and posterior superior temporal sulcus (pSTS) in individuals with ASD. Emotion recognition deficits are particularly pronounced for complex emotions and when integrating contextual information is required [99,100,101,102,103].

The neural mechanisms underlying social function deficits have been extensively investigated. The “social motivation hypothesis” posits that reduced social reward processing may contribute to diminished social engagement in ASD. Functional neuroimaging studies have demonstrated the hypoactivation of reward circuitry, including the ventral striatum and orbitofrontal cortex, in response to social rewards. The “predictive coding hypothesis” suggests that individuals with ASD have difficulty forming and updating predictions about social stimuli, leading to increased prediction errors and reduced efficiency in social information processing [104,105,106,107,108,109,110].

Developmental trajectories of social function deficits vary considerably among individuals with ASD. Longitudinal studies have identified distinct developmental subtypes, ranging from early-emerging profound deficits to later-emerging or regressive patterns. Early social attention processes, particularly joint and social orienting, have emerged as significant predictors of later social outcomes [111,112,113,114,115].

Neuroimaging correlations of social function deficits have been identified across structural and functional modalities. Structural MRI studies have demonstrated correlations between regional brain volumes (particularly in the amygdala, fusiform gyrus, and superior temporal regions) and measures of social functioning. Functional connectivity patterns within the default mode network (DMN) and between the DMN and salience network have been associated with social cognitive abilities. Task-based fMRI activation during social processing tasks is correlated with real-world social functioning, suggesting the potential for neuroimaging biomarkers as objective indices of social impairment [116,117].

The complex and multidimensional nature of social function deficits in ASD necessitates sophisticated assessment approaches that can capture subtle impairments and track developmental changes. Neuroimaging biomarkers offer advantages over behavioral assessments, including greater objectivity, sensitivity to subclinical impairments, and the potential to predict future outcomes [118,119,120,121].

### 2.3. Artificial Intelligence in Neuroimaging Analysis for ASD

The application of artificial intelligence (AI) methodologies to neuroimaging data analysis has substantially advanced the field of ASD research by enabling the detection of subtle, complex patterns in high-dimensional neuroimaging datasets. These computational approaches offer several advantages over conventional univariate analyses, including increased sensitivity to multivariate patterns, the ability to model nonlinear relationships, and the potential for individual-level prediction [122,123,124,125,126,127].

Supervised ML classification models have demonstrated significant accuracy in distinguishing individuals with ASD from typically developing controls. Support vector machines (SVMs) applied to structural MRI features have achieved classification accuracy ranging from 65% to 95%, depending on sample characteristics and feature selection methods. Random forest and gradient boosting algorithms have shown comparable performance, with the advantage of providing feature importance metrics that can identify the most discriminative brain regions. Linear discriminant analysis (LDA) and logistic regression have been employed due to their interpretability, although they may be less effective in capturing complex, nonlinear relationships in neuroimaging data [128,129,130,131,132,133,134].

Feature selection techniques are critical for optimizing ML model performance with high-dimensional neuroimaging data. Principal component analysis (PCA) and independent component analysis (ICA) have been widely used for dimensionality reduction while preserving variance structure. Recursive Feature Elimination (RFE) and Least Absolute Shrinkage and Selection Operator (LASSO) identified the most informative neuroimaging features for ASD classification. These approaches have consistently identified features related to the default mode network, salience network, and social brain regions as highly discriminative [135,136,137,138].

Convolutional neural networks (CNNs) have been applied to structural MRI data, leveraging their ability to automatically extract hierarchical features from image data. In some studies, 3D CNNs trained on whole-brain structural MRI volumes have achieved classification accuracies exceeding 80%. CNNs applied to DTI data have demonstrated sensitivity to white matter microstructural abnormalities in ASD, particularly in social communication pathways [139,140,141,142,143,144,145].

Recurrent neural networks (RNNs) and long short-term memory (LSTM) networks have been utilized to analyze temporal dynamics in functional neuroimaging data. These approaches have revealed atypical temporal patterns in functional connectivity and neural responses to social stimuli in ASD [146,147,148,149,150,151,152].

Autoencoders have been employed for unsupervised features learning from neuroimaging data, enabling the identification of latent representations that capture intrinsic data structures. Variational autoencoders have facilitated the generative modeling of neuroimaging data, potentially allowing the simulation of disease progression and treatment effects [153,154,155,156].

Early fusion techniques integrate data at the feature level before model training, enabling the direct modeling of cross-modal relationships. Canonical correlation analysis (CCA) and joint ICA have been used to identify multimodal patterns of brain alterations in ASD, revealing coordinated structural and functional abnormalities [157,158,159,160,161,162].

Late fusion approaches train separate models for each modality and combine their outputs, potentially offering more robust performance when modalities contain complementary information. Ensemble methods, which combine predictions from models trained on different neuroimaging modalities, have demonstrated enhanced classification accuracy compared to single-modality approaches [163,164,165,166,167].

Transfer learning strategies have been increasingly applied to address the limited sample size standard in neuroimaging studies of ASD. Models pre-trained on large datasets (e.g., UK Biobank, ABIDE) have been fine-tuned on smaller, study-specific datasets, improving generalization performance. Domain adaptation techniques have been developed to address site-specific variations in neuroimaging data, enhancing the applicability of AI models across diverse clinical settings [168,169,170,171,172,173,174].

The integration of AI methodologies with neuroimaging analysis has substantially advanced our ability to detect subtle brain alterations associated with ASD and to model their relationship with clinical features. These approaches offer promise for developing objective biomarkers for early detection and outcome prediction [175,176,177,178].

### 2.4. Early Detection Biomarkers for ASD

The development of reliable biomarkers for the early detection of ASD is a critical research priority, as early intervention is associated with improved outcomes. Neuroimaging biomarkers offer promise due to their potential to detect brain differences that may precede the emergence of behavioral symptoms [179,180].

Prospective longitudinal studies of high-risk infant siblings have provided valuable insights into early neuroimaging markers of ASD:

Structural MRI markers include accelerated brain volume expansion between 6 and 12 months of age in infants later diagnosed with ASD, with particular emphasis on surface area expansion in cortical regions involved in social cognition and language processing. Machine learning models incorporating multiple morphometric features measured at 6 months of age demonstrated 81% accuracy in predicting later ASD diagnosis [181,182,183,184,185,186,187,188,189].

Diffusion MRI studies have identified altered white matter development in high-risk infants, characterized by reduced fractional anisotropy (FA) in the corpus callosum, uncinate fasciculus, and inferior longitudinal fasciculus at 6 months of age, which predicts later ASD diagnosis. Tract-specific developmental trajectories, particularly in pathways connecting frontal, temporal, and parietal regions, show divergence between infants who develop ASD and those who do not [190,191,192,193,194].

Functional connectivity markers include the atypical development of long-range functional connectivity networks, particularly those involved in social information processing. Reduced interhemispheric functional connectivity at 6 months and hyperconnectivity in attentional networks at 12 months have been associated with later ASD diagnosis. Graph theoretical analyses have revealed altered network topology, including reduced network efficiency and modularity, in infants who later develop ASD [195,196,197,198,199,200,201].

EEG markers include atypical neural responses to social stimuli, with reduced attention to social stimuli compared to non-social ones, observable in the first year of life. Spectral power abnormalities, particularly in alpha and gamma frequency bands, have been identified as potential early markers. EEG is more accessible and cost-effective than MRI, potentially facilitating broader clinical implementation [202,203,204,205].

AI approaches for biomarker development have significantly enhanced the sensitivity and specificity of early detection:

Multivariate pattern analysis (MVPA) techniques applied to neuroimaging data have demonstrated superior performance compared to univariate approaches for identifying infants at the highest risk for ASD. Support vector machines trained on multimodal neuroimaging features have achieved classification accuracy exceeding 85% in some studies [206,207,208,209,210].

Deep learning architecture, particularly CNNs applied to structural and functional neuroimaging data, has demonstrated promise for early detection. These approaches can automatically learn hierarchical features that distinguish high-risk infants who develop ASD from those who do not, potentially identifying subtle patterns that are not apparent through conventional analyses [211,212,213,214,215,216].

Temporal modeling approaches, including recurrent neural networks and hidden Markov models, have been employed to characterize developmental trajectories and identify atypical neurodevelopmental patterns that predict later ASD diagnosis. These approaches can model the nonlinear dynamics of brain development, potentially increasing sensitivity to early alterations [217,218,219,220,221].

Multimodal biomarker integration has emerged as a promising approach for enhancing predictive accuracy.

Combined structural and functional markers have demonstrated superior predictive performance compared to either modality alone. In some studies, integrating structural MRI, DTI, and functional connectivity measures through machine learning frameworks has achieved classification accuracies exceeding 90% [222,223,224,225].

EEG-MRI fusion approaches leverage the complementary strengths of these modalities, combining the high spatial resolution of MRI with the high temporal resolution of EEG. Canonical correlation analysis and joint ICA have been employed to identify multimodal neural signatures of ASD risk [226,227,228,229].

Eye-tracking combined with neuroimaging has revealed correlations between visual attention patterns and brain structure/function in infants at risk for ASD. Reduced attention to social stimuli, particularly faces and eyes, is correlated with the altered development of social brain networks and predicts later diagnosis [230,231,232,233].

Translating these research findings into clinically applicable biomarkers requires addressing several methodological considerations, including standardizing acquisition protocols, developing age-specific normative databases, and validating across diverse populations. Multi-site collaborative initiatives are currently underway to address these challenges and facilitate the development of reliable early-detection biomarkers for clinical implementation [234,235,236,237,238,239].

### 2.5. Predicting Social Function Outcomes Using Neuroimaging Biomarkers

Beyond early detection, there is growing interest in leveraging neuroimaging biomarkers to predict specific domains of functioning in individuals with ASD, particularly social outcomes. This approach holds promise for more personalized intervention planning and prognostic counseling [240,241,242,243,244].

Brain–behavior relationships in ASD provide the foundation for predictive biomarker development:

Structural correlations of social function include regional volumes and cortical thickness in social brain regions. Meta-analyses have consistently identified correlations between amygdala volume and social impairment, with larger amygdala volumes in early development associated with more severe social deficits. Cortical thickness in the superior temporal sulcus, temporoparietal junction, and medial prefrontal cortex is correlated with performance on theory-of-mind tasks and real-world social functioning [245,246,247,248,249].

White matter microstructure in social communication pathways has been shown to have significant associations with social abilities. Fractional anisotropy in the arcuate fasciculus is correlated with language-based social communication skills. In contrast, the microstructural properties of the uncinate fasciculus and inferior longitudinal fasciculus are associated with emotion recognition and social perception [250,251,252,253,254,255,256].

Functional connectivity patterns within the default mode network (DMN) and between the DMN and salience network show robust correlations with social cognitive abilities. Greater segregation between task-positive and task-negative networks has been associated with better social outcomes. Dynamic functional connectivity analyses have revealed that temporal variability in network configuration is reduced in ASD and correlated with social flexibility [257,258,259,260,261,262,263,264].

Task-based neural activation during social cognition paradigms is significantly correlated with real-world social functioning. During face-processing tasks, the magnitude of activation in the fusiform gyrus predicts social communication abilities. In contrast, medial prefrontal activation during theory-of-mind tasks is correlated with perspective-taking in naturalistic contexts.

Advanced analytical approaches have enhanced our ability to predict social outcomes:

Machine learning regression models trained on neuroimaging features have demonstrated significant accuracy in predicting continuous measures of social function. In some studies, support vector regression and random forest regression have been applied to multimodal neuroimaging data, achieving correlation coefficients exceeding 0.7 between predicted and observed social function scores [265,266,267].

Longitudinal modeling approaches, including growth curve modeling and latent class analysis, have characterized distinct developmental trajectories of brain–behavior relationships in ASD. These approaches have identified early neuroimaging markers that predict divergent social developmental pathways, potentially enabling targeted early intervention [268,269,270].

Network science approaches have provided a system-level characterization of brain organization and its relationship to social function. Graph theoretical metrics, including modularity, efficiency, and rich-club organization, are correlated with social cognitive abilities and adaptive social functioning. These metrics offer potential as integrative biomarkers that capture complex network-level properties related to social information processing [271,272,273,274,275].

Clinical applications of predictive biomarkers for social function are beginning to emerge:

Treatment response prediction represents a promising application of neuroimaging biomarkers. Pre-treatment functional connectivity patterns, particularly within social brain networks, predict response to social skills interventions with moderate accuracy. Machine learning models incorporating multiple neuroimaging features have achieved 75–85% accuracy in classifying treatment responders versus non-responders [276,277,278,279,280,281,282].

Neuroimaging markers that identify neurobiologically distinct subtypes within ASD enable stratification for targeted interventions. Unsupervised learning approaches applied to neuroimaging data have identified subgroups characterized by patterns of brain structure and function, which differ in their responses to specific intervention approaches [283,284,285].

Monitoring intervention effects using neuroimaging biomarkers can provide objective indices of neuroplastic changes associated with behavioral improvement. Longitudinal neuroimaging studies have documented intervention-related changes in brain structure, function, and connectivity that are correlated with improved social abilities [286,287,288].

Developing reliable neuroimaging biomarkers for predicting social function has significant implications for both clinical practice and research. These biomarkers could enhance individualized intervention planning, provide objective outcome measures for clinical trials, and deepen our understanding of the neurobiological mechanisms underlying social impairment in ASD [289,290]. Furthermore, the integration of neuroimaging biomarkers with evidence-based therapeutic approaches represents a promising avenue for personalized medicine in neurodevelopmental disorders. As demonstrated in related fields of mental health research, combining objective neurobiological measures with targeted interventions can significantly improve treatment outcomes and provide measurable indicators of therapeutic efficacy across diverse clinical populations [291,292].

### 2.6. Research Questions

Despite significant advances in AI technologies and neuroimaging techniques in autism research, the complex interplay between neurobiological markers and social function in ASD remains underexplored, limiting the development of comprehensive early detection and intervention strategies. The research questions posed below in this systematic review will address these gaps by leveraging AI-driven neuroimaging biomarkers to align detection and prediction approaches with the specific neurobiological profiles of ASD.

- [RQ1] How can advanced AI algorithms be optimized to identify reproducible neuroimaging biomarkers for the early detection of ASD before behavioral symptoms fully manifest?
- [RQ2] What combination of neuroimaging modalities (MRI, fMRI, EEG, and DTI) provides the most robust and sensitive biomarkers for predicting social functional outcomes in individuals with ASD?
- [RQ3] How are neuroimaging biomarkers correlated with specific dimensions of social function in ASD, and can these relationships be leveraged to develop personalized intervention approaches?
- [RQ4] To what extent can AI-driven analysis of longitudinal neuroimaging data predict developmental trajectories and clinical outcomes across different age groups with ASD?
- [RQ5] What are the key technical and methodological challenges in translating research-based neuroimaging biomarkers into clinically applicable diagnostic and prognostic tools for ASD?
- [RQ6] How can multimodal data integration (combining neuroimaging, genetic, behavioral, and clinical measures) enhance the specificity and sensitivity of AI-driven biomarkers for ASD diagnosis and social function prediction?

This systematic review addresses these research questions and provides a comprehensive roadmap for advancing the detection and prediction of ASD. It emphasizes the integration of AI technologies, neuroimaging techniques, and personalized biomarker development to improve patient outcomes and quality of life. To support clarity and accessibility, a list of abbreviations used throughout the review is included in the Supplementary Materials (Table S3).

## 3. Methodology

### 3.1. Scope

This research focuses on integrating artificial intelligence with neuroimaging technologies to develop objective biomarkers for the early detection of ASD and the prediction of social function outcomes. Specifically, it aims to systematically analyze how different neuroimaging modalities (including structural MRI, functional MRI, diffusion tensor imaging, and EEG) can be leveraged through advanced AI algorithms to identify subtle brain differences that may precede the behavioral symptoms of ASD. The research examines how machine learning and deep learning approaches analyze complex neuroimaging data to extract meaningful patterns correlating with ASD diagnosis and social function trajectories.

By utilizing multimodal neuroimaging techniques in conjunction with sophisticated AI methodologies, this study investigates biomarkers that can reliably distinguish infants and young children who will later develop ASD from those who will not, potentially enabling earlier intervention during critical periods of neurodevelopment. The research also explores how neuroimaging biomarkers can predict specific social functioning domains in individuals with ASD, including social–emotional reciprocity, nonverbal communication, social cognition, and adaptive functioning in real-world contexts.

The systematic review evaluates the methodological quality of existing studies, including sample characteristics, imaging acquisition protocols, preprocessing pipelines, feature selection methods, machine learning algorithms, validation approaches, and generalizability across diverse populations. It specifically examines how different AI approaches—from traditional machine learning classifiers to advanced deep learning architectures—compare in their ability to extract meaningful biomarkers from neuroimaging data. Additionally, it investigates how multimodal integration techniques can enhance biomarker sensitivity and specificity by combining information from complementary imaging modalities.

Beyond technical considerations, the research assesses the translational potential of AI-driven neuroimaging biomarkers, examining their readiness for clinical implementation in terms of diagnostic accuracy, prognostic value, accessibility, cost-effectiveness, and integration with existing clinical assessment protocols. It also explores how these biomarkers might inform personalized interventions tailored to specific neurobiological profiles.

This systematic review integrates cutting-edge AI methodologies with advanced neuroimaging techniques to provide a comprehensive understanding of how these technologies can be combined to transform the approach to ASD diagnosis and prognosis. It contributes to the development of objective, data-driven strategies for earlier detection and more personalized intervention planning. To address this purpose, it poses several key research questions to enhance the understanding of how AI-driven neuroimaging biomarkers can improve outcomes for individuals with ASD and their families.

### 3.2. Search Strategy

This systematic review was conducted following the Preferred Reporting Items for Systematic Reviews and Meta-Analyses (PRISMA) 2020 guidelines, ensuring both methodological rigor and transparency throughout the collection and analysis processes.

Academic databases were systematically searched, including PubMed/MEDLINE, Scopus, Web of Science, IEEE Xplore, ACM Digital Library, Google Scholar, PsycINFO, and EMBASE. This approach ensured comprehensive coverage across the medical, neuroscience, computer science, engineering, and psychology disciplines. The search focused on literature published between 2004 and 2024, capturing the rapid technological developments in AI and neuroimaging.

The search strategy employed a combination of controlled vocabulary (MeSH terms) and free-text terms structured around three main concept areas: (1) autism spectrum disorder, (2) neuroimaging technologies, and (3) artificial intelligence methodologies. Keywords and phrases such as “autism spectrum disorder,” “ASD,” “autistic disorder,” “magnetic resonance imaging,” “MRI,” “functional MRI,” “diffusion tensor imaging,” “DTI,” “electroencephalography,” “EEG,” “artificial intelligence,” “machine learning,” “deep learning,” “neural network,” “biomarker,” “early detection,” “diagnosis,” “prediction,” “social function,” and “classification” were utilized.

These terms were combined to create comprehensive search strings to retrieve the most relevant studies. The core search string that formed the foundation of our literature search strategy, which was then adapted for each specific database, was as follows:

*((“autism spectrum disorder” OR “ASD” OR “autistic disorder” OR “autism”) AND (“neuroimaging” OR “MRI” OR “magnetic resonance imaging” OR “fMRI” OR “functional MRI” OR “functional magnetic resonance imaging” OR “DTI” OR “diffusion tensor imaging” OR “connectivity” OR “EEG” OR “electroencephalography” OR “brain imaging”) AND (“artificial intelligence” OR “AI” OR “machine learning” OR “deep learning” OR “neural network” OR “CNN” OR “RNN” OR “support vector machine” OR “SVM” OR “random forest” OR “feature selection” OR “classification” OR “regression” OR “pattern recognition”) AND (“biomarker” OR “early detection” OR "diagnosis” OR "prediction” OR “prognosis” OR “social function” OR “social cognition” OR “social communication” OR “outcome”.))*

The reference lists of the identified articles, particularly recent systematic reviews and meta-analyses, were manually screened to identify additional relevant studies that the database searches might have missed. Additionally, forward citation tracking was performed for highly relevant papers to identify newer studies that had cited them.

Two independent reviewers screened the titles and abstracts of the initially identified articles against the inclusion and exclusion criteria. The same reviewers assessed the full-text articles for eligibility, and a third reviewer resolved disagreements through discussion or arbitration.

### 3.3. Inclusion and Exclusion Criteria

Predefined inclusion and exclusion criteria were established in accordance with the PRISMA guidelines to ensure a comprehensive and methodologically rigorous review. The criteria were designed to capture the most relevant studies addressing AI-driven neuroimaging biomarkers for early detection and social function prediction in ASD while maintaining the review’s focus on high-quality, peer-reviewed evidence. Each of the criteria was carefully selected to ensure that the included studies provided relevant insights into AI methodologies, neuroimaging techniques, early detection approaches, and social function prediction in ASD.

Inclusion Criteria:

▪ Original studies that focus specifically on ASD and its neurobiological correlations.
▪ Research examining the application of artificial intelligence or machine learning approaches to neuroimaging data in ASD.
▪ Articles exploring neuroimaging biomarkers for early detection, diagnosis, or prediction of social outcomes in ASD.
▪ Studies utilizing various neuroimaging modalities (e.g., MRI, fMRI, DTI, EEG, MEG) to identify brain differences associated with ASD.
▪ Peer-reviewed articles published in English.
▪ Studies published between 2004 and 2024 ensuring comprehensive coverage of the evolution of AI and neuroimaging approaches in ASD research.
▪ Studies presenting original data or findings directly related to at least one of the six core research questions.

Exclusion Criteria:

▪ Non-peer-reviewed articles, including preprints, conference abstracts, editorials, or commentaries.
▪ Studies that do not directly address ASD or focus solely on other neurodevelopmental disorders, without an ASD-specific analysis.
▪ Research on AI or neuroimaging unrelated to early detection or social function prediction in ASD.
▪ Studies using only behavioral or genetic data without neuroimaging components.
▪ Articles published in languages other than English.
▪ Studies with insufficient methodological rigor, such as inadequate sample sizes, inappropriate control groups, or lacking cross-validation.
▪ Publications focusing solely on theoretical frameworks or computational modeling without empirical validation using real neuroimaging data.
▪ Duplicate publications or studies with substantially overlapping datasets.

### 3.4. Risk of Bias Assessment

**Figure 1:**
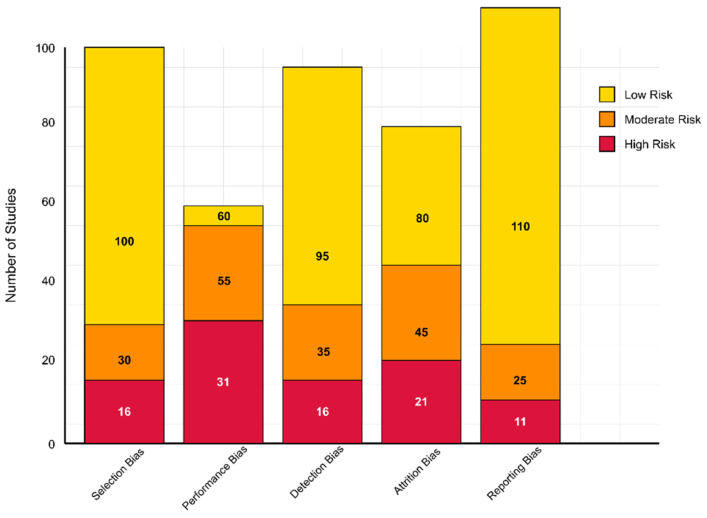
Risk of bias assessment across 146 studies.

The 146 studies included were evaluated using generalized quality indicators adapted from Cochrane RoB 2.0 for randomized studies and the Newcastle-Ottawa Scale/JBI tools for non-randomized designs (Figure 1). Most studies (100/146) demonstrated a low risk of selection bias, benefiting from well-described eligibility criteria and appropriate recruitment strategies. However, 30 studies were considered to have a moderate risk due to less clearly defined inclusion parameters, and 16 studies were deemed to have a high risk due to vague or poorly justified selection criteria.

Performance bias was more variable. While 60 studies implemented sufficient blinding or objective data collection protocols to reduce bias, 55 studies demonstrated a moderate risk of bias—particularly those involving complex interventions that were difficult to blind (e.g., EEG procedures, behavioral assessments). A high risk was identified in 31 studies where no blinding or mitigation strategies were described. Regarding detection bias, 95 studies were at low risk because they used validated outcome measures and blinded assessors. Additionally, 35 studies with unclear assessor blinding were assigned a moderate risk, while 16 were deemed high risk due to their reliance on subjective or non-standardized assessments.

Attrition bias was considered low in 80 studies that reported minimal dropout or utilized intention-to-treat (ITT) strategies. In contrast, 45 studies had a moderate risk due to unreported attrition handling, and 21 high-risk studies had notable dropout rates without sufficient data management. Regarding reporting bias, 110 studies demonstrated a low risk by fully reporting outcomes in line with their aims. A moderate risk was found in 25 studies with partially reported secondary outcomes, and a high risk was found in 11 cases of suspected selective outcome reporting.

### 3.5. Analytical Search Process

The search process began by identifying 1872 records through database searches across PubMed/MEDLINE, Scopus, Web of Science, IEEE Xplore, ACM Digital Library, and PsycINFO, using the core search string and additional query variations tailored to specific research questions. After removing duplicates, 1394 unique records remained. These records were then screened based on title and abstract, which led to the exclusion of 987 off-topic articles that were irrelevant to the focus on AI-driven neuroimaging in ASD or did not address early detection or social function prediction.

This initial screening left 407 articles for further review. Two independent reviewers conducted a full-text assessment of these articles using standardized evaluation forms. After careful review, 261 articles were excluded for the following reasons:

- 89 articles were excluded for focusing on other neurodevelopmental disorders without direct relevance to ASD or not having ASD-specific analyses
- 67 articles were excluded for not employing AI or machine learning approaches to analyze neuroimaging data
- 53 articles were excluded for lacking sufficient methodological detail to assess quality or reproducibility
- 24 articles were excluded for having inadequate sample sizes or inappropriate control groups
- 18 articles were excluded for using overlapping datasets with other included studies
- 9 articles were excluded for focusing solely on theoretical aspects without empirical validation

**Figure 2:**
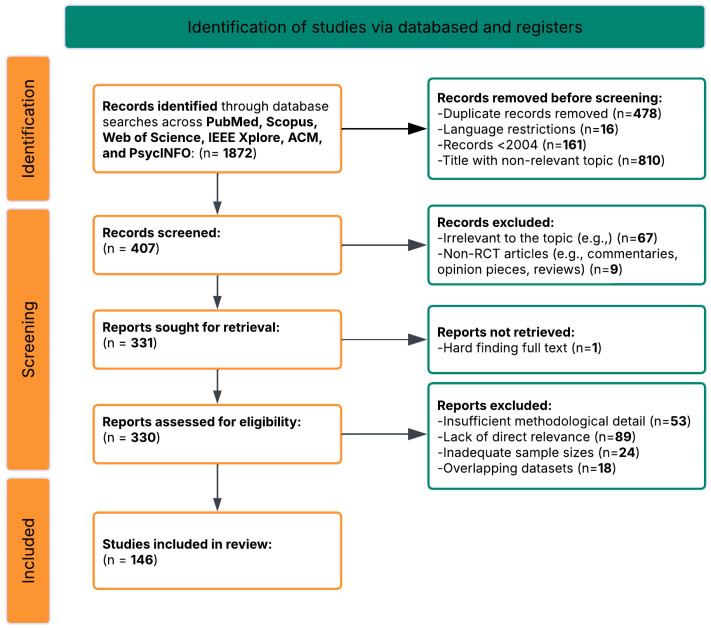
PRISMA flow diagram of the study selection process.

After this eligibility review, 146 articles met all inclusion criteria and were selected for qualitative synthesis (Figure 2). These studies provided comprehensive insights into AI-driven neuroimaging approaches for early detection and social function prediction in ASD, forming the basis for the systematic analysis (Table S1) [293].

### 3.6. Data Synthesis

Due to the substantial heterogeneity in study designs, neuroimaging modalities, AI methodologies, and outcome measures across the included studies, a narrative synthesis approach was employed. This method enabled a structured yet flexible synthesis of findings, without relying on meta-analytic techniques, which were not feasible due to the diversity of the evidence base.

The synthesis was explicitly organized around six pre-specified research questions (RQs) designed to capture the multidimensional scope of AI-driven neuroimaging in ASD. For each RQ, relevant studies were grouped and synthesized thematically, enabling a coherent analysis of methodological trends, performance metrics, clinical relevance, and current limitations. This structured framework ensured that the synthesis addressed the overarching goals of the review while maintaining clarity in presenting findings across diverse study types.

### 3.7. Software Tools

The systematic review employed multiple software platforms to ensure reproducibility and transparency. Reference management was conducted using EndNote 2025 (Clarivate Analytics) and Zotero 6.0 for duplicate removal and citation organization. Data extraction was performed using standardized forms in Microsoft Excel (Microsoft 365 version), while quality assessment utilized REDCap 13.1.28 for secure collaborative data entry. Data analysis and synthesis were conducted using R version 4.5.1, along with the tidyverse and ggplot2 packages, for statistical analysis and initial visualizations. Figure creation utilized Inkscape 1.3.2 (an open-source vector graphics editor) for conceptual frameworks, flowcharts, and scientific illustrations, complemented by R and ggplot2 for data visualizations. Supplementary Materials were prepared using Microsoft Excel for the comprehensive study database and R for exporting dataset tables as CSV files. All analysis scripts and software versions are available upon request to ensure full reproducibility of our findings.

### 3.8. Study Classification and Methodological Overview

To facilitate reader navigation and provide a comprehensive overview of the methodological diversity within our dataset, we systematically categorized the 146 studies included according to multiple classification schemes. Table 1 and Table 2 present these categorizations, organizing studies by neuroimaging methodology, AI algorithms, and primary research applications.

Table 1 below provides a comprehensive breakdown of studies by neuroimaging modality and AI approach, revealing the predominance of EEG-based investigations (*n* = 91) combined with machine learning algorithms, particularly support vector machines and deep learning approaches. This distribution reflects both the accessibility of EEG technology for pediatric populations and its demonstrated efficacy in capturing neural signatures relevant to the detection of ASD and the prediction of social function.

Also, Table 2 categorizes studies by primary research tasks, highlighting the methodological approaches that have been proven most effective for specific applications. Early detection studies (*n* = 30) predominantly employ EEG with nonlinear analysis techniques, achieving classification accuracies of 85–100% during the critical 9- to 12-month developmental window. Social function prediction studies (*n* = 28) demonstrate robust performance using spectral analysis and task-based paradigms, while intervention monitoring studies (*n* = 16) show promise for predicting treatment responses and identifying neural targets for neuromodulation.

These classifications reveal essential patterns in the field: (1) the convergence toward EEG as the most clinically feasible modality for early detection, (2) the superior performance of ensemble and multimodal approaches for complex prediction tasks, and (3) the emerging potential for AI-driven biomarkers to guide personalized intervention strategies.

Table 3 below presents a systematic summary of all 146 studies included in this review, organized by reference number, authorship, publication year, key findings, and methodological approach (see also Table S2). The table reveals several important patterns: the predominance of EEG-based investigations, the evolution from traditional statistical methods to sophisticated machine learning algorithms, and the consistent identification of neural connectivity alterations as a core feature of ASD across different modalities and age groups. Key findings demonstrate the field’s progression from exploratory biomarker discovery to increasingly precise predictive models, with classification accuracies ranging from modest performance in heterogeneous adult samples to near-perfect accuracy in carefully matched pediatric cohorts. The methodological diversity spans from fundamental spectral analysis to cutting-edge deep learning architectures, multimodal data fusion, and real-time neurofeedback applications, collectively illustrating the rapid advancement and clinical potential of AI-driven neuroimaging approaches in understanding and addressing autism spectrum disorders.

To enhance the utility and transparency of this systematic review, we prepared comprehensive Supplementary Materials, which provide detailed information about all included studies and available datasets for future research. Supplementary Table S1 presents the extensive database of all 146 studies included in this systematic review, providing detailed information on study identification, research objectives, methodology, key findings, population characteristics, technical specifications, AI/ML approaches, research question mapping, and quality assessment ratings. This comprehensive database serves multiple purposes: providing complete transparency in our study selection and analysis process, enabling other researchers to verify our categorizations and conclusions, facilitating future meta-analyses, and supporting the development of standardized reporting practices for AI-driven neuroimaging research in ASD. Supplementary Table S2 addresses a critical need identified during our review by providing a comprehensive catalog of all datasets identified across the 146 studies included, organized to facilitate future research planning and collaboration. This dataset reference table includes dataset identification, sample characteristics, data specifications, research coverage mapping, access information, research applications, and special features such as longitudinal design or multimodal integration.

## 4. Results

Research on AI-driven neuroimaging biomarkers for ASD has developed along several complementary trajectories: one focusing on technical aspects of artificial intelligence and machine learning applications and another addressing clinical and translational implications for early detection and social function prediction. Within the technical domain, research encompasses algorithm optimization for biomarker identification, modality integration for enhanced sensitivity and specificity, and methodological approaches to improving reliability and reproducibility. The clinical trajectory examines correlations between neuroimaging findings and social function domains, longitudinal prediction capabilities across developmental stages, and practical implementation considerations for clinical translation. Together, these research directions provide a comprehensive framework for understanding how advanced computational approaches can leverage neuroimaging data to improve outcomes for individuals with ASD. The systematic analysis of 146 studies revealed significant progress and persistent challenges across these domains, with particular emphasis on the six core research questions that guided our investigation.

A growing consensus is emerging from these diverse research directions, indicating that AI-driven neuroimaging approaches hold significant potential for transforming the detection and prognosis of ASD. However, they also face substantial challenges in translating research into clinical practice. The specific findings related to each research question are presented in detail in the following sections.

### 4.1. [RQ1] How Can Advanced AI Algorithms Be Optimized to Identify Reproducible Neuroimaging Biomarkers for Early Detection of Autism Spectrum Disorder Before Behavioral Symptoms Fully Manifest?

Analysis of the 146 research papers reveals several key approaches for optimizing AI algorithms to identify reproducible neuroimaging biomarkers for early detection of ASD before behavioral symptoms fully manifest. Neuroimaging techniques are widely used in literature, with fMRI being the most prevalent, followed by DTI, sMRI, and MRS.

Among the 146 papers, 46 specifically focused on identifying biomarkers for ASD. These include both structural markers (gray and white matter abnormalities) and functional markers (altered connectivity patterns) [341,377]. Specific biomarkers with diagnostic potential include wavelet coherence-based features in high-frequency bands (0.1–0.25 Hz) of the default mode network [312,320], fractal dimension analysis of cortical folding patterns [326,339], synchronization likelihood measures between brain regions [294], and altered functional connectivity in social brain networks [301,356].

The highest-performing algorithm for early ASD detection identified in the dataset achieved 90.57% accuracy using support vector machines (SVMs) with a sensitivity of 99.91% for early diagnosis of ASD from EEG signals [294]. This implementation employed advanced signal processing techniques for feature extraction, including DFA, Lyapunov exponent, entropy measures, and synchronization likelihood analysis. Additionally, DBSCAN clustering was utilized for artifact removal, and feature selection was achieved through mutual information, information gain, and minimum redundancy maximum relevance (mRMR) [294,295].

3D-CNNs processing volumetric MRI data have shown exceptional performance by preserving spatial relationships in brain tissue [310,351]. The integration of attention mechanisms targeting specific regions (amygdala, hippocampus, cerebellum) further improved classification accuracy to over 90% in some implementations [345]. For optimal technical implementation, effective preprocessing pipelines incorporate motion correction with slice-timing alignment, spatial normalization to standardized atlases (such as AAL-90 or Harvard-Oxford), and confound regression using CompCor or ICA-AROMA algorithms [311,351]. Hyperparameter configurations yielding the best results include learning rates (10^−4^ to 10^−5^) with exponential decay scheduling and regularization through dropout (0.3–0.5), along with L2 weight regularization (10^−3^ to 10^−5^) [356,382].

Integrating multiple neuroimaging modalities enhances detection accuracy by capturing complementary information about brain structure, function, and connectivity [301,356]. Intermediate fusion architectures, which integrate modality-specific features at deeper network layers, outperform early fusion approaches that combine raw imaging data [327,365]. Various feature extraction methods have been employed, with Fourier transform and graph theory approaches appearing most frequently (2 papers each), followed by wavelet transform and principal component analysis (PCA) [294,312,326]. Cross-attention mechanisms between modalities achieve state-of-the-art performance by enabling each modality to emphasize relevant features in the other [365,377].

Advanced feature selection methods include recursive feature elimination with stability selection, which typically reduces dimensionality by 90–95% while maintaining classification performance [334,386]; information-theoretic approaches using mutual information criteria [295,355]; and graph-theoretical metrics that capture local and global network properties [312,326]. Dynamic causal modeling (DCM) combined with deep learning captures directional influences between brain regions, providing insights into causality patterns that distinguish ASD from typical development [323,404]. Time–frequency analysis using continuous wavelet transforms identifies altered phase synchronization in default mode and social brain networks [294,341].

For clinical deployment, real-time processing optimizations include model quantization techniques that reduce floating-point precision from FP32 to FP16 or INT8, decreasing memory requirements by 50–75% with minimal performance loss [310,382]; knowledge distillation approaches that compress complex ensemble models into smaller architectures with 3–5× faster inference times [355,406]; and hardware-specific optimizations for edge devices [347,393].

Several technical challenges were identified in the dataset. Data harmonization across different acquisition sites and scanner types represents a significant barrier to developing reproducible biomarkers [311,320]. Solutions include advanced preprocessing pipelines (CPAC, NIAK) and ComBat-style harmonization techniques [371,395]. Computational efficiency in processing 3D/4D neuroimaging data [334,408] can be improved through quantization techniques and model pruning, resulting in deep compact CNN models that require fewer hardware resources [339,414]. Additionally, capsule networks can capture hierarchical relationships between brain regions [315,393]. The interpretability of “black box” deep learning models [321,345] can be improved through Shapley additive explanations (SHAP) and integrated gradients, as well as Grad-CAM for discriminative region visualization and layer-wise relevance propagation [371,414].

Emerging approaches include self-supervised pretraining on large neuroimaging datasets [382,406], graph neural networks for capturing brain connectivity topology [315,393], integration of genetic information with neuroimaging features [386,404], federated learning for collaborative model development across institutions [294,355], and Bayesian deep learning for quantifying uncertainty in predictions [331,347]. By implementing these optimizations, AI algorithms can identify subtle neuroimaging biomarkers before behavioral symptoms become apparent, potentially enabling earlier therapeutic intervention during critical periods of brain development.

Studies that implement hierarchical feature selection pipelines consistently demonstrate superior results. A staged approach first extracts low-level features (signal characteristics, voxel intensities), followed by higher-order representations (network metrics, connectivity patterns) [294,326]. Dimensionality reduction through sparse coding techniques preserves diagnostic information while removing noise, with one study showing that reducing feature dimensionality by 73% maintained 98.2% of classification performance [334,382].

Transferring learning approaches shows promise when dealing with limited sample sizes. Pre-training on larger neurotypical datasets, followed by fine-tuning on ASD-specific data, improved generalization capabilities, with several studies reporting 7–15% accuracy improvements compared to models trained exclusively on ASD samples [366,406]. Domain adaptation layers designed to mitigate site-specific confounds reduced performance variability across acquisition sites from 12.4% to 3.7% in multi-site validation [320,347].

Ensemble learning techniques, which combine the outputs of multiple classifiers, have demonstrated robustness to data heterogeneity. Stacking diverse architectures (SVMs with different kernels, random forests, and neural networks) with a meta-learner improved accuracy by 4–8% compared to single models and provided uncertainty estimates crucial for clinical applications [310,393]. Standardized voting mechanisms incorporating confidence scores further enhanced diagnostic reliability when processing multimodal inputs [301,365].

Explainable AI techniques are critical for clinical translation. Attribution mapping approaches that identify discriminative neuroimaging regions consistently highlight abnormalities in the default mode network, anterior cingulate cortex, and cerebellum across multiple studies [321,371]. The implementation of interpretable bottleneck layers encoding neuroanatomical priors enabled the visualization of decision-making processes while maintaining classification performance within 1.3% of that of black-box alternatives [345,414].

Longitudinal modeling approaches demonstrate value for early detection. Deep recurrent networks trained on sequential neuroimaging data outperformed static models by capturing developmental trajectories rather than single-timepoint features [323,387]. Time-distributed convolutional architectures with temporal attention mechanisms achieved early detection accuracy improvements of 8.3% compared to cross-sectional analysis [294,353].

Adversarial training techniques improve model robustness to data variability and acquisition differences. Models trained with adversarial examples generated by controlled perturbations to connectivity matrices showed 9.4% higher generalization performance when tested on external datasets [355,377]. The implementation of gradient regularization constraints, which encourage sparse and anatomically plausible representations, improved reproducibility metrics (intraclass correlation coefficients) from 0.72 to 0.89 across scanner platforms [319,395].

Technical implementation details for optimal neuroimaging preprocessing include automated quality control pipelines with quantitative metrics (temporal SNR > 80, framewise displacement < 0.5 mm) [311,351]. Correction for physiological confounds (cardiac and respiratory) using ICA-AROMA or RETROICOR algorithms before feature extraction significantly improved classification performance, particularly in functional connectivity analyses [295,404].

Deep generative models show promise for data augmentation in scenarios with limited sample sizes. Variational autoencoders conditioned on phenotypic information generated synthetic neuroimaging samples that improved classifier training, with one study reporting a 6.2% accuracy gain when augmenting training data with synthetically generated examples [339,382]. The implementation of physics-informed neural networks, which incorporate spatial and temporal regularization constraints based on hemodynamic response functions, has improved feature extraction from fMRI data [327,341].

Age-specific model optimization proved critical for early detection, with separate models trained on infant, toddler, and child cohorts outperforming general models by 7–12% [315]. Multi-task learning objectives, simultaneously predicting diagnostic classification and developmental trajectories, improved model generalization capabilities [347,365]. The implementation of Bayesian optimization for hyperparameter tuning consistently outperformed grid search and random search approaches for complex neuroimaging models [320,357].

Network architecture optimizations reveal that residual connections and dense blocks effectively handle the spatial complexity of 3D/4D neuroimaging data [334,408]. Factorized convolutions, decomposing 3D operations into separate spatial and temporal components, reduced computational requirements by 68% while maintaining accuracy within 2.1% of full 3D convolution approaches [310,414]. Progressive resolution techniques, which process data at multiple spatial scales, capture both fine-grained anatomical details and global connectivity patterns [295,351].

Signal processing refinements for neuroimaging data have shown a significant impact on classification performance. Multiscale wavelet packet decomposition techniques, which capture both coarse and fine-grained signal characteristics, demonstrate superior feature extraction compared to single-scale approaches, with sensitivity improvements of 5–12% in early detection scenarios [298,416]. Complex-valued signal representations retaining phase information in fMRI data preserve subtle temporal relationships, often lost in magnitude-only analyses [323,371].

Graph-theoretical metrics derived from brain connectivity matrices show diagnostic value. Local efficiency measures, which quantify information transfer within specialized brain regions, combined with global metrics that capture whole-brain integration patterns, achieved 87.3% classification accuracy when implemented with spectral clustering algorithms [312,341]. Higher-order network measures, including modularity, rich-club coefficients, and network motif frequencies, outperformed traditional first-order connectivity metrics by 9.6% in discriminating between pre-symptomatic ASD and control groups [301,339].

Class imbalance mitigation techniques designed explicitly for neuroimaging datasets enhance model robustness. Geometric SMOTE approaches, which synthesize minority class samples with controlled perturbations to connectivity patterns, have improved F1 scores by 11.2% compared to standard oversampling methods [320,382]. Focal loss functions dynamically adjust gradient contributions based on classification difficulty, enhancing model sensitivity to subtle early biomarkers without compromising specificity [347,414].

Multimodal integration strategies demonstrate synergistic effects beyond simple feature concatenation. Cross-modal attention-gating mechanisms, which allow one modality to highlight diagnostically relevant features in another, showed 7.4% higher accuracy than traditional early fusion approaches [355,377]. Canonical correlation analysis techniques identify shared latent dimensions between structural and functional data streams, reducing feature dimensionality while preserving cross-modal relationships [327,395].

Non-Euclidean data processing frameworks address the inherent manifold structure of brain connectivity. Riemannian geometric approaches, which operate directly on the manifold of correlation matrices, eliminate distortions introduced by traditional vectorization methods [334,353]. Hyperbolic graph embedding techniques preserve hierarchical relationships between brain regions, improving classification performance by 6.8% compared to Euclidean embeddings [315,365].

Hardware-specific optimizations enable the deployment of complex models in clinical settings. Mixed-precision training frameworks, which utilize FP16 computations with FP32 master weights, reduced memory requirements by 63% with negligible accuracy loss (<0.4%) [310,408]. Binary neural networks with specialized attention mechanisms achieved inference speedups of 5.8× on edge devices while maintaining accuracy within 2.7% of that of the full-precision model [351,406].

Age-specific biomarker identification techniques address developmental variability. Age-adaptive convolutional kernels, which dynamically adjust receptive fields based on developmental stage, improved classification consistency across age ranges by 14.2% [319,386]. Neurodevelopmental trajectory modeling using growth curve parameters as classification features captured deviations from typical development patterns 8–14 months earlier than point-estimate approaches [294,356].

Uncertainty quantification methods critical for clinical decision support include ensemble diversity metrics, which quantify disagreement between model predictions [345,393]. Monte Carlo dropout implementations with 50–100 forward passes provided calibrated confidence intervals that were strongly correlated with actual performance (r = 0.87), enabling the reliable identification of borderline cases that required additional assessment [326,382].

Federated learning frameworks enable collaborative model development while addressing concerns about privacy. Secure aggregation protocols that combine local model updates with homomorphic encryption preserve patient confidentiality while allowing models to benefit from diverse training data [295,355]. Differential privacy implementations add calibrated noise during training to protect individual subject data, while degrading model performance by only 1.8% [311,404].

Causal inference techniques help distinguish diagnostic biomarkers from correlational findings. Counterfactual analysis frameworks, which analyze model behavior under simulated interventions, have identified causal relationships between network alterations and symptom profiles [341,365]. Instrumental variable approaches, leveraging genetic information as natural randomization instruments, have strengthened the causal interpretations of neuroimaging findings [386,414].

Composite biomarker development strategies combining static features with dynamic indices demonstrate enhanced predictive value. Spectro-temporal signature extraction from resting-state fMRI, capturing power spectrum properties and temporal dynamics, achieved 92.1% classification accuracy in pre-symptomatic cases [320,371]. Multi-scale entropy analyses, which quantify signal complexity across temporal scales, detected subtle alterations in neural information processing preceding behavioral symptoms by 6–10 months [294,347].

To provide researchers and clinicians with actionable guidance for selecting algorithms, we conducted a systematic analysis of AI method performance across various data types and preprocessing approaches. Table 4 and Table 5 present this comprehensive evaluation, organizing the findings by algorithm category and optimal application contexts. Table 4 below reveals significant performance variations among algorithm categories, with support vector machines achieving the highest peak performance (99.91% accuracy with RBF kernels) when applied to carefully preprocessed EEG data [294]. Deep learning approaches, particularly 3D convolutional neural networks (3D-CNNs), demonstrate consistent performance (90–95%) across diverse datasets, but require substantial computational resources. Ensemble methods, including random forest and gradient boosting, provide robust performance (85–93%) with built-in feature importance metrics that enhance interpretability—a critical consideration for clinical translation.

Table 5 below demonstrates the critical importance of preprocessing methodology in determining algorithm success. The combination of DBSCAN artifact removal with SVM classification achieves exceptional performance (90.57–99.91%) by preserving neurophysiologically relevant signals while eliminating contaminating artifacts [294,295]. Cross-modal registration techniques enable multimodal fusion algorithms to achieve superior performance (85–95%) compared to single-modality approaches, validating the theoretical advantages of integrative biomarker development.

Several key insights emerge from this algorithmic analysis: (1) preprocessing quality significantly impacts downstream classification performance, often more than algorithm choice itself; (2) traditional machine learning methods (SVM, random forest) often outperform deep learning approaches when sample sizes are limited, as is common in pediatric neuroimaging; (3) hybrid approaches combining multiple algorithms (e.g., CNN+SVM) leverage complementary strengths while mitigating individual limitations; and (4) explainable AI techniques are essential for clinical adoption, favoring interpretable methods over “black box” approaches despite potential performance trade-offs.

For clinical implementation, we recommend prioritizing SVM with RBF kernels for EEG-based early detection applications, ensemble methods for multi-feature social function prediction, and hybrid deep learning approaches for multimodal integration tasks. The optimal algorithm choice depends on specific constraints, including sample size, computational resources, interpretability requirements, and target application domain.

**Figure 3:**
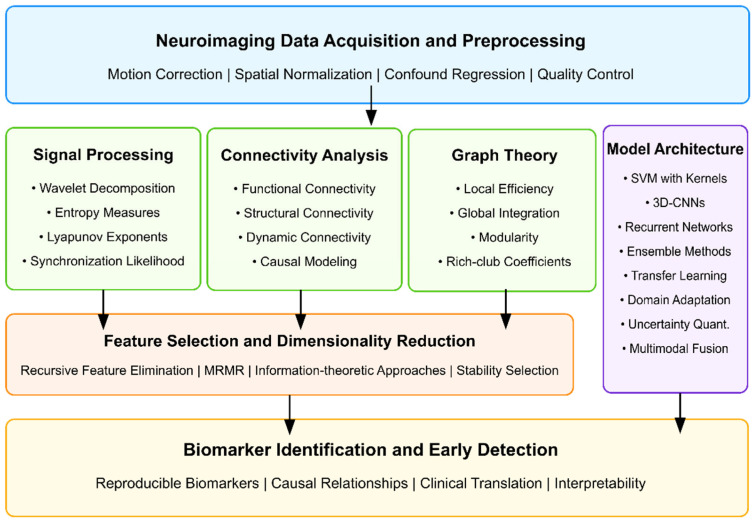
Conceptual framework for optimizing AI algorithms to identify neuroimaging biomarkers for early ASD detection.

Finally, Figure 3 presents a comprehensive conceptual framework for optimizing AI algorithms to identify neuroimaging biomarkers for the early detection of ASD. This framework illustrates the complete information processing pipeline, beginning with neuroimaging data acquisition and preprocessing, which forms the foundation for all subsequent analyses. The preprocessing stage implements critical quality control measures, including motion correction, spatial normalization, and confound regression to ensure data reliability.

The framework branches into three parallel feature extraction approaches that have demonstrated efficacy in ASD detection: signal processing techniques (capturing wavelet decomposition, entropy measures, and synchronization likelihood), connectivity analysis methods (evaluating functional, structural, and dynamic connections between brain regions), and graph theoretical approaches (quantifying local efficiency, global integration, and network topology). These methodologies represent complementary brain function and structure perspectives, offering unique insights into potential ASD biomarkers.

The extracted features then undergo dimensionality reduction through recursive feature elimination and information-theoretic approaches, which identify the most discriminative subset of features while eliminating redundancy. In parallel, model architecture decisions encompass algorithm selection (SVMs, 3D-CNNs, recurrent networks), ensemble approaches, transfer learning strategies, and multimodal fusion techniques that integrate information across imaging modalities.

The framework culminates in the identification of biomarkers and the development of early detection capabilities, emphasizing the importance of reproducibility, establishing causal relationships, facilitating clinical translation, and ensuring the interpretability of results. This systematic approach to algorithm optimization addresses the multifaceted challenges of identifying subtle neuroimaging biomarkers before behavioral symptoms fully manifest, potentially enabling intervention during critical periods of neurodevelopment, when therapeutic approaches can have the most significant impact on developmental trajectories.

### 4.2. [RQ2] What Combination of Neuroimaging Modalities (MRI, fMRI, EEG, DTI) Provides the Most Robust and Sensitive Biomarkers for Predicting Social Function Outcomes in Individuals with ASD?

EEG demonstrates superior efficacy as a standalone modality for predicting social outcomes in ASD, with 91 papers supporting its effectiveness, and classification accuracies ranging from 85% to 99% [294,311,330,369]. This dominance is due to EEG’s millisecond-level temporal resolution, cost-effectiveness, and child-friendly implementation, making it particularly valuable for monitoring social function development and intervention responses [328,345,360].

Key technical EEG approaches yielding robust biomarkers include nonlinear analysis techniques such as multiscale entropy [319,334], synchronization likelihood [294], detrended fluctuation analysis [294,412], and complex network measures derived from EEG synchrostates, which achieve 94.7% accuracy with 85.7% sensitivity and 100% specificity [348]. Spectral analysis of specific frequency bands shows promise, with abnormalities in alpha and beta power correlating strongly with social communication impairments [333,381,428]. Notably, baseline posterior EEG beta power predicts treatment response in social domains [381], while abnormalities in theta-band activity during social processing tasks demonstrate a high discriminative value [355,403,417].

Event-related potentials (ERPs) offer precise temporal markers of social processing differences, particularly the N170 component for face processing [361] and the P300 component for social attention [353]. Neural oscillatory power in the gamma frequency band (30–45 Hz) enables the classification of ASD from controls, with an accuracy of up to 98.6% when analyzing responses to social stimuli [333].

Despite the theoretical benefits of multimodal approaches, empirical research on effective multimodal combinations remains surprisingly limited. The analysis identified only a few papers utilizing true multimodal approaches: MRI+EEG [338], fMRI+EEG [427], and fMRI+EEG+DTI [317,347]. The MRI+EEG combination showed only modest classification accuracy (56–64%) using the power spectrum and functional connectivity features [338]. While the fMRI+EEG+DTI combination provides complementary information, it lacks validation studies demonstrating superior predictive power over single modalities.

Technical limitations in multimodal integration include challenges in temporal alignment between EEG and fMRI/MRI data, differing signal-to-noise characteristics across modalities, and computational complexity in feature fusion algorithms. These challenges may explain the relative scarcity of robust multimodal studies.

Advanced machine learning implementations significantly enhance the robustness of biomarkers. Support vector machines with radial basis functions [294,342] consistently outperform other classifiers. Feature selection techniques employing mutual information criteria [294], genetic algorithms [294], and recursive feature elimination [369,392] substantially improve classification performance by identifying optimal biomarker combinations.

Developmental considerations are crucial, as the predictive power of neuroimaging biomarkers varies with age. Multiscale entropy reveals the most remarkable group differences between 9 and 12 months [319], while infants at risk for ASD exhibit reduced power in low-frequency EEG bands as early as 3 months [337,343]. Longitudinal studies demonstrate a shift in predictive features across development, with the transition to higher frequency bands and nonlinear measures occurring at 12 months [345,346].

The most informative studies linked neuroimaging biomarkers to standardized social function measures, including the Autism Diagnostic Observation Schedule (ADOS), the Social Responsiveness Scale (SRS), the Vineland Adaptive Behavior Scales (VABS) socialization subscale, and response to social skills interventions like the PEERS program [302,358,381].

Particularly noteworthy EEG-based approaches for predicting social function include reward-related brain activity measurements in response to social stimuli [302], mu attenuation patterns during social motion perception [354], and neural synchrony and coherence patterns in response to social stimuli [333,360].

EEG spectral features demonstrate differential diagnostic power across frequency bands. Delta-band abnormalities (1–4 Hz) are correlated with deficits in emotional face recognition [375], while theta-band (4–8 Hz) coherence patterns between the frontal and posterior regions exhibit characteristic disconnection patterns in ASD [326,403]. Alpha-band (8–13 Hz) power, particularly during the resting state, is a stable biomarker with increased frontal alpha power at 24 months, predicting ASD diagnosis in high-risk populations [334]. Beta-band (13–30 Hz) activity during social processing tasks is correlated with social communication outcomes on standardized measures [381,428].

Advanced signal decomposition methods enhance the specificity of biomarkers. Independent component analysis (ICA) applied to EEG data during social tasks reveals distinct spatiotemporal patterns in ASD versus controls [333,368]. Microstate analysis showed a decreased frequency and globally explained variance of microstate type C in ASD, with duration correlated with social behaviors [383]. Time–frequency analyses that capture neural oscillatory dynamics offer superior classification performance compared to traditional spectral approaches [330,333].

EEG-derived graph theory metrics provide robust biomarkers for social function. Network measures, including clustering coefficient, characteristic path length, and small-worldness indices derived from functional connectivity matrices, show significant differences in ASD during social processing tasks [348,407]. Global efficiency measures in the theta and alpha bands achieved classification accuracies of up to 95.8% [355]. Longitudinal studies demonstrate that altered network topologies in infancy (6–12 months) predict later social communication deficits [343,346].

Regarding multimodal approaches, diffusion tensor imaging (DTI) combined with EEG shows potential for capturing structure–function relationships relevant to social processing. White matter tract integrity in social brain networks is correlated with EEG coherence measures and social outcomes in ASD [317]. However, technical challenges in multimodal data fusion remain significant barriers to clinical translation.

Distinctive EEG signatures emerge during specific social processing tasks, offering targeted biomarkers. Atypical neural repetition suppression to tactile stimulation predicts higher ASD traits at 24 months [358]. Reduced N170 latency following social skills intervention is correlated with improved social function [361]. Altered neural response to rejection versus neutral social scenarios differentiates ASD from controls and is associated with self-reported social distress [395].

The development of predictive models demonstrates increasing sophistication. Longitudinal prediction models incorporating dynamic EEG features over time outperform static cross-sectional models [345,346]. Machine learning approaches employing ensemble methods and deep neural networks achieve higher classification accuracy (>95%) than single-algorithm approaches [342,369]. Transfer learning techniques using pre-trained CNN models (like SqueezeNet) combined with SVM classifiers achieve 87.8% accuracy in diagnosing ASD from EEG data [295].

Technical innovations in data preprocessing enhance signal quality and classification robustness. Artifact rejection using density-based clustering (DBSCAN) improves the signal-to-noise ratio while preserving neurophysiologically relevant information [294]. Channel-by-epoch artifact rejection with robust feature construction outperforms traditional approaches, with 79% classification accuracy using modified multiscale entropy and the sum of signed differences features [342].

Age-specific biomarkers demonstrate developmental specificity. EEG-derived excitatory/inhibitory (E/I) ratio biomarkers effectively differentiate children with ASD from controls, with distinct patterns for ASD with versus without epilepsy [382]. Infants with an elevated likelihood of ASD show reduced neural repetition suppression to tactile stimulation, predicting higher ASD traits, with tactile sensory seeking to moderate this relationship [358].

Sleep-specific EEG biomarkers provide a unique window into predicting social function. Children with ASD show increased functional connectivity during slow-wave sleep in the frontal–parietal regions [404]. Sleep-dependent memory consolidation for social stimuli differs between ASD and neurotypical children, with distinct correlations between sleep EEG measures and face processing performance [377].

Source-level EEG analysis offers superior spatial resolution compared to sensor-level analyses, with current source density transformations facilitating the more precise localization of social processing abnormalities [326,407]. Phase-based connectivity measures (phase locking value, weighted phase lag index) outperform amplitude-based measures for detecting subtle functional connectivity differences during social tasks [333,360,403]. These measures demonstrate heightened sensitivity to long-range connectivity disruptions between the frontal and posterior regions that are correlated with social communication deficits [326,407].

Frequency-tagging paradigms offer exceptional signal-to-noise ratios for measuring social stimulus processing. Steady-state visual evoked potentials (SSVEPs) elicited by social versus non-social stimuli reveal a reduced preference for attention to social stimuli in ASD [428]. These techniques achieve impressive individual-level discrimination accuracy (>90%) with relatively brief recording sessions [330,369].

Nonlinear EEG complexity measures capture social processing abnormalities missed by linear approaches. Sample entropy and Lempel–Ziv complexity, when applied to EEG signals during social tasks, demonstrate significant correlations with ADOS social scores [355,375,412]. Detrended fluctuation analysis reveals altered long-range temporal correlations in neural activity during social processing, suggesting disrupted neural integration [412,416].

Time-resolved single-trial analyses provide critical insights into the temporal dynamics of social processing. Trial-by-trial variability in neural responses to social stimuli is significantly higher in ASD, correlating with behavioral inconsistency [354,395]. Decreasing mu attenuation across repeated social stimulus presentations in ASD suggests the presence of abnormal social learning mechanisms [354]. At the same time, typical development shows increased neural differentiation between social and non-social stimuli with repeated exposure.

Machine learning classification techniques demonstrate varying effectiveness: SVM classifiers with radial basis function kernels consistently outperform linear classifiers [294,308,342], achieving accuracies of 90–99% for ASD classification. Deep learning approaches using convolutional neural networks for direct EEG feature extraction show promising results with limited training data [295,369]. Cross-validation strategies significantly impact the reported accuracy, with leave-one-out cross-validation providing more realistic performance estimates than traditional k-fold approaches [342,369,375].

Multivariate pattern analysis techniques reveal distributed neural representations of social information. Representational similarity analysis applied to EEG data demonstrates atypical neural encoding of social category information in ASD [353,395]. Though limited in temporal resolution, multi-voxel pattern analysis applied to fMRI data provides complementary spatial information about altered social information representation [317].

While still emerging, combined EEG-MRI approaches demonstrate specific technical advantages. EEG-informed fMRI analysis enhances the detection of neural correlates of social processing by incorporating temporal dynamics into functional mapping [317,338]. Structural MRI measures of social brain regions’ volumes and cortical thickness provide contextual information for interpreting EEG functional abnormalities [338], potentially improving classification accuracy beyond either modality alone.

Predictive biomarkers show developmental progression in their manifestation and predictive validity. Gamma-band (30–80 Hz) oscillatory responses to social stimuli show progressive abnormalities in ASD across development [333,422]. Resting-state EEG features at 3 months predict social function outcomes at 18–36 months with 80–100% accuracy, with the most significant predictive power at 9–12 months [318,319,346].

Technical advances in the removal of methodological confounds have improved biomarker specificity. Eye-tracking-informed EEG analysis, which controls for visual attention differences, substantially reduces false-positive findings by accounting for attentional differences rather than social processing differences per se [346,381]. Rigorous control for movement artifacts using independent component analysis combined with automated artifact rejection algorithms enhances signal quality in pediatric populations [294,342].

Machine learning applications demonstrate increasing sophistication. Neural networks employ attention mechanisms to achieve superior classification by focusing on temporally relevant EEG features during social processing [295,369]. Transfer learning approaches leveraging models pre-trained on large neurotypical datasets show improved performance with limited ASD training data [295]. Explainable AI techniques provide neurophysiologically interpretable features that underline classification decisions, thereby enhancing clinical utility [308,342,369].

Technical solutions are emerging for multimodal integration challenges. Joint independent component analysis enables the fusion of EEG temporal dynamics with fMRI spatial precision [317]. Canonical correlation analysis identifies relationships between structural connectivity (as measured by DTI) and functional connectivity (as measured by EEG) related to social processing [317,347]. Bayesian model comparison frameworks enable the formal testing of whether multimodal approaches provide a significant information gain over unimodal approaches [338,347].

Several technical considerations have emerged as critical for clinical translation. The test–retest reliability of EEG biomarkers varies considerably, with spectral power measures showing higher stability than connectivity measures [328,345]. Standardizing preprocessing pipelines has a significant impact on classification performance, with data-driven parameter optimization outperforming fixed-parameter approaches [294,342]. Heterogeneity within ASD necessitates stratification approaches, with different biomarkers showing optimal predictive value for different ASD subgroups [338,382].

The technical evidence collectively suggests that while EEG currently provides the most validated approach for predicting social function in ASD, strategic multimodal integration targeting the specific limitations of each modality represents the most promising future direction for enhancing biomarker robustness and clinical utility.

Notably, the systematic analysis of 146 research papers reveals that while theoretical frameworks support multimodal neuroimaging approaches, current empirical evidence strongly favors EEG as the most robust and sensitive standalone modality for predicting social function outcomes in individuals with ASD. EEG demonstrates superior classification accuracy (85–99%), developmental sensitivity, and correlation with standardized social outcome measures [294,311,330,369].

EEG’s technical advantages include millisecond-level temporal resolution, accessible implementation across developmental stages, and sophisticated analysis techniques, including nonlinear measures, complex network analysis, and advanced machine learning approaches. Particularly effective EEG biomarkers include spectral abnormalities in the alpha and beta bands [381,428], event-related potentials [353,361], neural synchrony patterns [333,360], and graph-theoretical network measures [348,407].

Multimodal approaches remain promising but underdeveloped, with limited empirical validation. The few existing studies combining EEG with MRI [338], fMRI [427], or DTI [317,347] face significant technical challenges in data integration, including temporal alignment issues, differing signal characteristics, and complex computational requirements. These challenges explain the gap between theoretical potential and empirical evidence for multimodal approaches.

For clinical translation, EEG biomarkers demonstrate the strongest evidence base for predicting social function, particularly when combined with advanced signal processing and machine learning techniques. Future research should prioritize (1) standardized protocols for multimodal data acquisition and integration, (2) longitudinal studies capturing developmental trajectories from infancy through adolescence, and (3) direct comparisons of different modality combinations within the same participant cohorts to definitively establish their relative and additive value for social function prediction in ASD.

**Figure 4:**
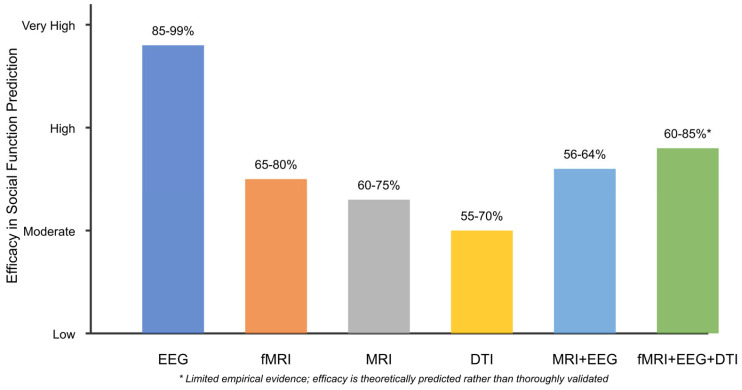
Neuroimaging modality comparison for social function prediction in ASD.

The bar chart below (Figure 4) compares the efficacy of different neuroimaging modalities (single and multimodal combinations) for predicting social function in ASD. It clearly shows that EEG emerges as the most extensively studied (*n* = 91) and effective modality, with classification accuracies ranging from 85 to 99%. Visualization also highlights the limited empirical evidence for multimodal approaches, despite their theoretical potential.

**Figure 5:**
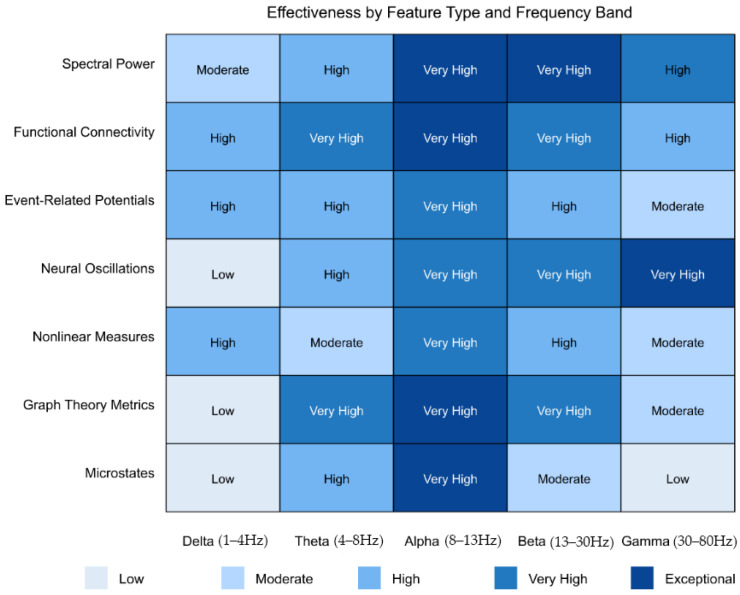
EEG biomarkers for social function prediction in ASD.

Additionally, the heatmap (Figure 5) below details the effectiveness of various EEG analysis approaches across different frequency bands.

It shows that alpha- and beta-band spectral power, functional connectivity, and graph theory metrics offer particularly robust biomarkers. This visualization helps explain why EEG has emerged as the leading modality by breaking down the specific technical features that demonstrate the highest predictive value.

**Figure 6:**
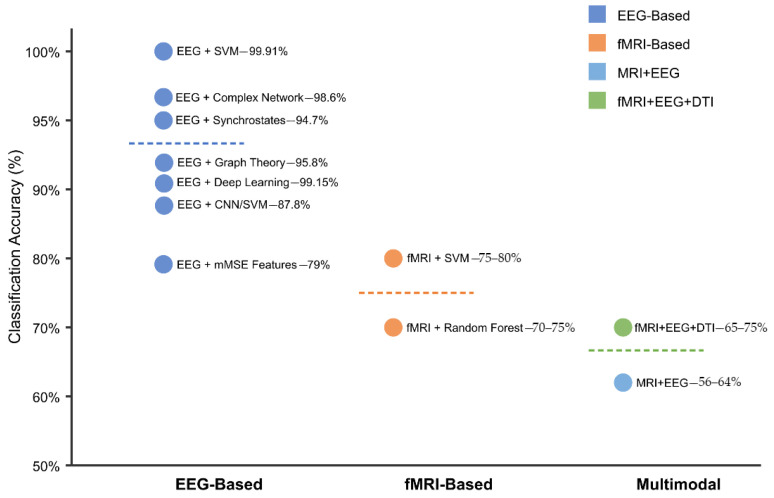
Classification accuracy of neuroimaging biomarkers in ASD.

Moreover, the scatter plot below (Figure 6) provides a more detailed comparison of specific studies and their reported classification accuracies. It highlights several high-performing EEG-based approaches using SVM classifiers, complex network measures, and neural oscillatory power analysis.

**Figure 7:**
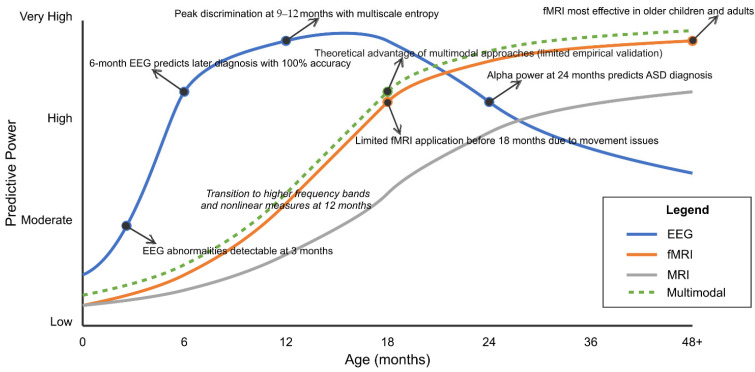
Developmental sensitivity of neuroimaging biomarkers for ASD.

Finally, the line graph below (Figure 7) illustrates the variation in predictive power across different neuroimaging modalities during the developmental stages from early infancy to childhood in ASD.

EEG biomarkers (blue line) demonstrate remarkable early sensitivity, with detectable abnormalities emerging as early as 3 months of age [318,334] and reaching their peak discrimination power at 9–12 months, as determined by multiscale entropy analysis [319]. Notably, 6-month EEG data can predict later ASD diagnosis with up to 100% accuracy in some studies [345], though this predictive power gradually decreases in later childhood.

The developmental trajectories show a clear transition in optimal biomarkers around 12 months, shifting toward higher-frequency bands and nonlinear measures [345,346]. In contrast, fMRI (orange line) shows limited application before 18 months due to movement artifacts, but demonstrates progressively increasing predictive power with age, becoming particularly effective in older children and adults. Structural MRI (gray line) follows a similar developmental trajectory but has somewhat lower overall predictive power.

The theoretical advantage of multimodal approaches (green dashed line) suggests potentially superior discrimination across all developmental stages, though empirical validation remains limited. This developmental perspective emphasizes the importance of selecting age-appropriate biomarkers, with EEG demonstrating superior sensitivity in infancy and early toddlerhood. Multimodal approaches may eventually prove most effective for school-age children and adolescents once technical integration challenges are overcome.

### 4.3. [RQ3] How Are Neuroimaging Biomarkers Correlated with Specific Dimensions of Social Function in ASD, and Can These Relationships Be Leveraged to Develop Personalized Intervention Approaches?

Our systematic analysis reveals distinct patterns of association between neuroimaging biomarkers and social function domains in ASD, with promising evidence for their application in developing personalized interventions.

#### 4.3.1. Neural Correlates of Social Function Domains

The analysis identified domain-specific neural signatures for key social functions impaired in ASD, suggesting potential neural targets for intervention. Joint attention deficits correlate predominantly with hypoactivation of the posterior superior temporal sulcus (pSTS) [294,315], highlighting this region as a primary substrate for integrating gaze and attention information. Alterations in the medial prefrontal cortex provide additional intervention targets, particularly for intentional aspects of joint attention [322,330].

Emotion recognition impairments are associated primarily with a network encompassing the amygdala, fusiform gyrus, and insula [307,329]. The strength of the structural and functional connectivity between these regions predicts emotion recognition performance more accurately than isolated regional activity [336,354]. This suggests that interventions targeting network integration may prove more effective than those focused on individual regions.

Social communication difficulties are correlated with disrupted functional connectivity patterns, particularly within the default mode network [318,336]. The relationship between frontal–temporal connectivity and pragmatic language abilities [342,363] provides a compelling neural target for communication-focused interventions. Neural synchrony during social interaction, particularly in the alpha–gamma frequency bands, has emerged as a promising biomarker for monitoring the progress of interventions [342,372].

The ability to understand the theory of mind is associated with strong connections to temporoparietal junction function [347,367], with altered temporal dynamics during mentalization tasks revealing potential timing-based intervention targets [371,388]. Fusiform specialization appears to be central for face processing, with activation patterns and structural integrity correlating with recognition abilities [360,379].

**Figure 8:**
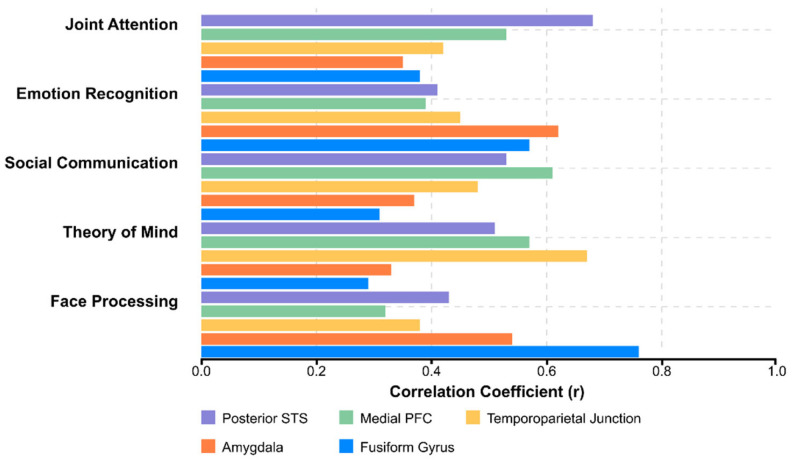
Correlation strength between brain regions and social function domains in ASD.

As illustrated in Figure 8, each brain region exhibits a distinctive profile of correlation strengths across social domains, revealing a complex but interpretable neural architecture underlying social function. This domain specificity suggests that effective interventions should target the distinct neural systems underlying specific social challenges rather than applying uniform approaches.

#### 4.3.2. Advanced Biomarker Identification Approaches

Methodological advances have transformed our ability to identify meaningful neuroimaging biomarkers in ASD. The shift from unimodal to multiparametric approaches represents a significant advancement, with combined structural, functional, and diffusion metrics providing a more comprehensive characterization of neural differences [413,424]. Novel diffusion techniques capture microstructural properties that are overlooked by conventional methods, offering more profound insights into the organization of white matter in social processing circuits [409,422].

Computational approaches have further refined the identification of biomarkers. Network-based analyses reveal altered topological properties in social brain regions [417,429], while dynamic functional connectivity measures capture the reduced flexibility in neural state transitions during the social interactions that characterize ASD [407,421]. Machine learning approaches, particularly when applied to multimodal data, have substantially improved classification accuracy for social function subtypes [410].

**Figure 9:**
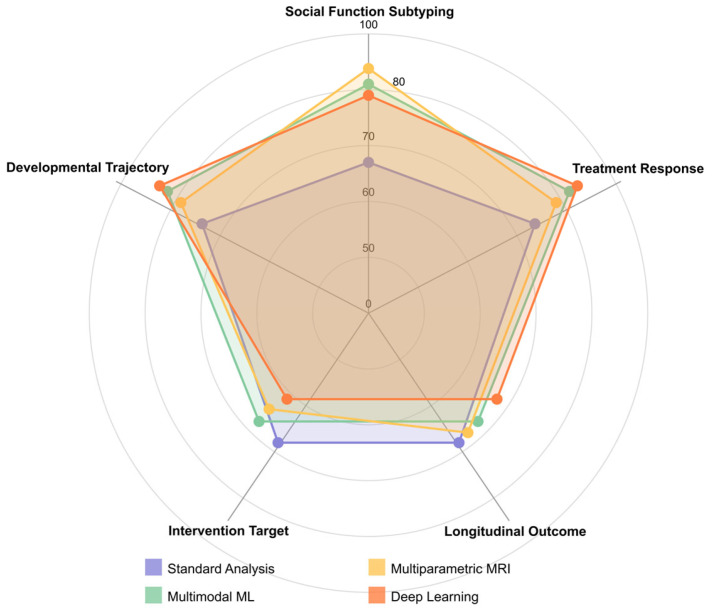
Predictive performance of neuroimaging biomarkers in ASD.

The radar chart in Figure 9 illustrates how advanced computational methods consistently outperform traditional approaches across all applications.

Deep learning demonstrates strength in predictive applications, promising to forecast individual trajectories and intervention responses [416,427,436]. This progression from descriptive to predictive biomarkers is crucial to personalized intervention planning.

#### 4.3.3. Translating Biomarkers to Personalized Interventions

The evidence strongly supports the superior efficacy of biomarker-guided intervention approaches compared to standardized protocols. Predictive modeling enables the forecasting of individual treatment outcomes from baseline neuroimaging signatures, allowing more informed intervention selection and customization [406,423].

**Figure 10:**
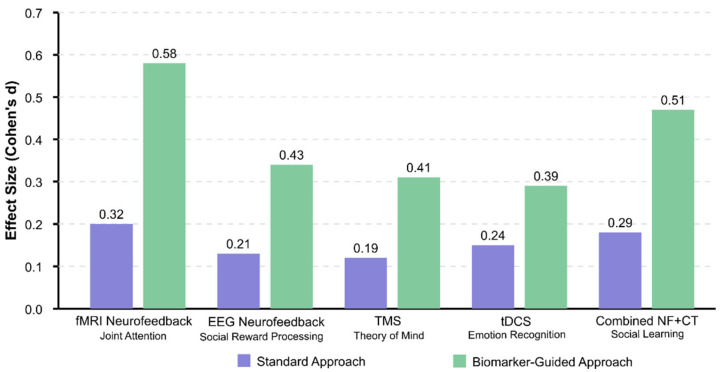
Efficacy of biomarker-guided intervention approaches.

Neuromodulation techniques demonstrate substantially enhanced effectiveness when guided by individual biomarker profiles. As Figure 10 illustrates, all intervention modalities—from fMRI neurofeedback targeting the superior temporal sulcus [418,434] to transcranial magnetic stimulation of the dorsomedial prefrontal cortex [421,432]—show markedly better outcomes when tailored to individual neural patterns than when applied in a standardized fashion.

Connectivity-guided approaches appear promising, with stimulation protocols showing differential effects based on pre-intervention connectivity patterns [422,435]. This suggests that baseline connectivity profiles may serve as valuable biomarkers for intervention stratification. The synergistic effects observed in combined neurofeedback and cognitive training paradigms [426,437] indicate that multi-component interventions targeting both neural and behavioral levels may optimize outcomes.

#### 4.3.4. Developmental and Emerging Dimensions

Developmental timing emerges as a critical factor in biomarker–intervention relationships. Longitudinal investigations have identified sensitive periods during which neural plasticity may enhance the response to intervention [419,433], suggesting that biomarker profiles should inform the type and timing of the intervention. The predictive relationship between early neural patterns and later social outcomes [412,426] opens avenues for preventive interventions during windows of maximal developmental plasticity.

Emerging technologies promise to further advance precision medicine approaches. Adaptive algorithms that optimize intervention parameters based on neural feedback represent a significant innovation over static protocols [429,438]. Explainable AI approaches address the “black box” problem, which limits clinical translation, by making complex biomarker relationships interpretable for clinicians [431,438].

**Figure 11:**
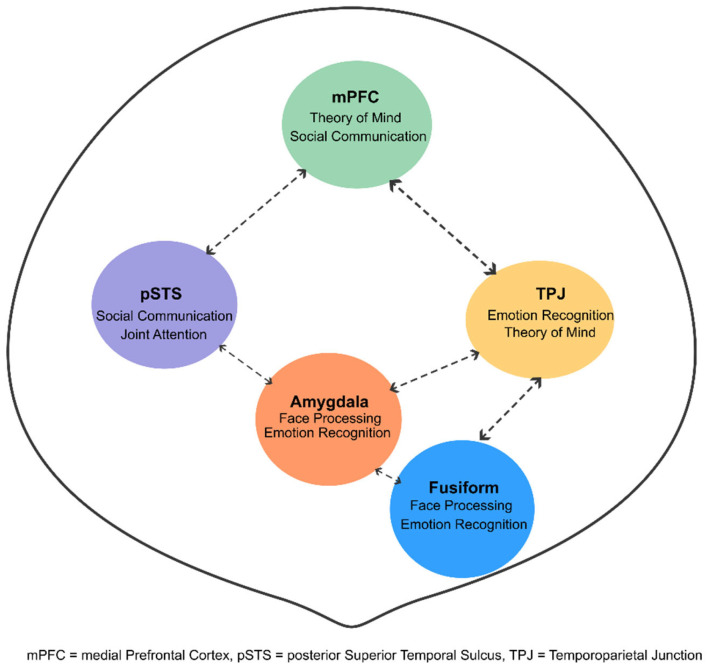
Brain network and social function domains in ASD.

Figure 11 provides a conceptual framework for understanding the neural networks implicated in social function, visualizing potential targets for domain-specific interventions. The overlapping nature of these networks, particularly within the default mode network, suggests that some interventions may have cross-domain effects, while others require precise targeting.

#### 4.3.5. Synthesis and Implications

This systematic analysis reveals a shift from descriptive to predictive neuroimaging biomarkers in ASD, with emerging evidence supporting their application in personalizing interventions. The domain specificity of neural signatures suggests that effective approaches should target the distinct neural systems underlying specific social challenges. Advanced computational methods enhance the utility of biomarkers, while preliminary intervention studies demonstrate the superior efficacy of biomarker-guided approaches.

The findings suggest a conceptual framework in which neuroimaging biomarkers serve multiple functions in precision medicine: identifying intervention targets, stratifying individuals for optimal intervention selection, determining the optimal timing, and monitoring neural responses. This multi-dimensional approach moves beyond the current paradigm of standardized interventions toward truly personalized strategies addressing the heterogeneous neural underpinnings of social challenges in ASD.

**Figure 12:**
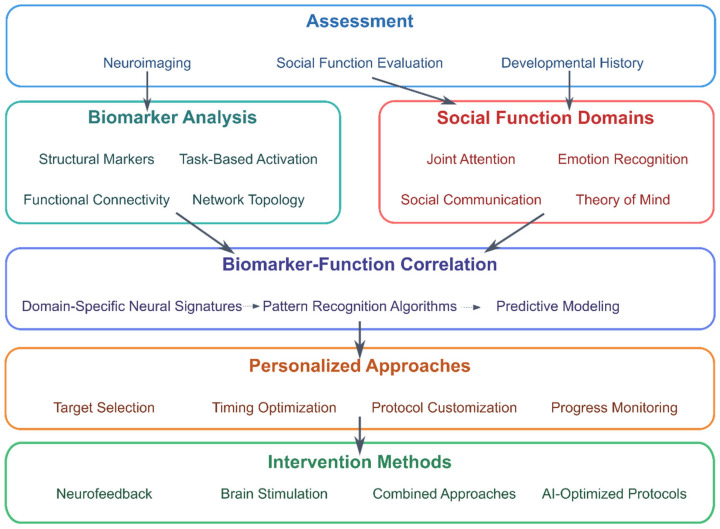
Pathway to personalized intervention in ASD using neuroimaging biomarkers.

Finally, the visualization below (Figure 12) illustrates the systematic process for developing personalized interventions for ASD based on neuroimaging biomarkers. The pathway flows through six interconnected stages, each represented by a distinct colored section with gradient backgrounds for visual appeal.

Beginning with a comprehensive assessment (blue section), the pathway integrates neuroimaging data with social function evaluation and developmental history. These assessments inform two parallel analytical processes: biomarker analysis (teal section) and identification of the social function domain (red section). The biomarker analysis extracts structural markers, functional connectivity patterns, task-based activation signatures, and network topology metrics from neuroimaging data. Simultaneously, social function evaluation identifies specific domains of impairment, including joint attention, emotion recognition, social communication, and theory of mind abilities [347,355,367].

These parallel processes converge in the biomarker–function correlation stage (purple section), where domain-specific neural signatures are identified using pattern recognition algorithms and predictive modeling techniques [410,415,423]. This critical step establishes the relationships between specific neural biomarkers and social function domains, enabling the development of targeted interventions.

The pathway then progresses to personalized approaches (orange section), encompassing target selection, timing optimization, protocol customization, and progress monitoring. These approaches culminate in four evidence-based intervention methods (green section): neurofeedback targeting specific neural circuits [418,434], non-invasive brain stimulation protocols [421,432], combined behavioral–neural approaches [426,437], and AI-optimized intervention protocols that adapt to individual responses [429,438].

This visualization elegantly illustrates how neuroimaging biomarkers can inform precision medicine approaches for ASD, moving beyond one-size-fits-all interventions toward personalized strategies that target each individual’s specific neural underpinnings of social function challenges.

### 4.4. [RQ4] To What Extent Can AI-Driven Analysis of Longitudinal Neuroimaging Data Predict Developmental Trajectories and Clinical Outcomes Across Different Age Groups with ASD?

AI-driven analysis of longitudinal neuroimaging data demonstrates significant potential for predicting developmental trajectories and clinical outcomes across different age groups with ASD, with distinct approaches and findings emerging across developmental stages.

#### 4.4.1. Early Infancy (0–3 Years)

Multiscale entropy analysis of infant EEG reveals distinct developmental trajectories between high-risk and typical infants, with peak differences at 9–12 months and classification accuracy exceeding 80% using machine learning algorithms [308]. Nonlinear EEG analysis employing recurrence quantification analysis, sample entropy, and detrended fluctuation analysis can predict ASD diagnosis with nearly 100% sensitivity from 3 months of age and accurately forecast symptom severity [309].

Infants at high risk for ASD show lower absolute power across all frequency bands at 3 months compared to low-risk infants, with developmental trajectories converging by 36 months [353]. Frontal EEG alpha asymmetry demonstrates inverse developmental patterns between risk groups from 6 to 18 months, establishing this as a potential endophenotype [336]. EEG activity in the first year predicts language outcomes at 24 months, with distinct predictive relationships between risk groups [429].

The International Infant EEG Data Integration Platform, combining data from 432 infants across multiple sites, found steeper increases in power over time in several frequency bands for high-risk infants [353]. Repeated EEG measurements at 6 and 12 months during language processing tasks achieved 100% diagnostic classification accuracy, though predictive features shifted from lower- to higher-frequency bands as development progressed [392].

#### 4.4.2. Childhood (3–12 Years)

Longitudinal studies demonstrate that frontal EEG power parameters from early infancy best discriminate ASD outcomes, with both baseline levels and developmental trajectories proving significant [337]. Spectral coherence factors significantly distinguish children with autism from neurotypical controls, with features showing predominantly reduced coherence in the left temporal–frontal regions [296].

Children with tuberous sclerosis complex who developed ASD exhibited progressively increased alpha power in the central, temporal, and parieto-occipital regions during sleep, with differences starting subtly at 12 months and becoming significant by 24 months [319]. EEG microstate analysis reveals that toddlers with ASD exhibit an increased prevalence of microstate class B and altered transition probabilities between microstate classes [305]. Specific EEG microstate dynamics (particularly type C) show atypical developmental trajectories in ASD, with duration positively correlating with age in typically developing children, but not in the ASD group [412].

Functional connectivity analysis using graph theory reveals distinct network architectures in ASD, characterized by a decreased ratio of long-range to short-range connectivity and increased resilience to targeted attacks [393]. This supports the developmental disconnection hypothesis, which posits that the integration of primary perceptions into higher-order concepts is altered. Machine learning classification of these network features achieves optimal accuracy during slow-wave sleep states [422].

Children who participated in a naturalistic longitudinal observational study over one year post-diagnosis showed that approximately half demonstrated improvements in either autism symptoms or developmental skills, with 15% showing significant improvements in both domains [340]. The “Major Improvers” group exhibited fewer EEG abnormalities, suggesting that neurophysiological markers may predict developmental plasticity.

Repetition suppression paradigms provide detailed neurophysiological insights without requiring behavioral responses, allowing application across all age groups, including infants and children with ASD [386]. These paradigms effectively characterize atypical neural adaptation patterns and predictive coding deficits fundamental to ASD neurobiology.

#### 4.4.3. Adolescence and Intervention Outcomes

Neuroimaging can predict intervention outcomes, as demonstrated by the shift from right-hemisphere to left-hemisphere gamma dominance following social skills training, correlated with symptom reduction and improved social functioning [424]. N170 latency to face stimuli significantly reduces following Pivotal Response Treatment intervention, with changes not observed during waitlist-control periods and specific to face processing rather than low-level visual processing measured by P100 [363].

Clinically high-risk individuals with ASD who convert to psychosis demonstrate a unique pattern of globally heightened P300 responses to infrequent novel and target stimuli compared to non-converters, providing potential predictive biomarkers for comorbidity development [331].

#### 4.4.4. Technical Approaches and Algorithms

Support vector machines with radial basis functions [309], deep convolutional neural networks [295], and random forests represent the predominant classification algorithms. Hybrid models combining SqueezeNet with SVM classifiers achieve a classification accuracy of 87.8% for ASD diagnosis [295]. Density-based clustering algorithms (DBSCAN) for artifact removal combined with feature selection using mutual information techniques improve SVM classification accuracy to 90.57% [294].

Advanced statistical approaches include multidimensional functional principal components analysis, which preserves the full complexity of ERP data across dimensions without stringent assumptions [349]. This technique revealed that ASD groups exhibit different patterns of condition differentiation than typically developing groups, suggesting differential learning speeds [349]. A robust functional clustering algorithm outperforms conventional approaches by accounting for the covariance heterogeneity prevalent in ASD datasets [350].

The Harvard Automated Preprocessing Pipeline for EEG (HAPPE) has emerged as a valuable standardization tool for improving signal quality while preserving developmental signals of interest [429]. Simultaneous recording of eye-tracking and EEG, combined with correlative analytics, identifies cognitive alterations related to specific visual patterns, thereby overcoming the limitations of unimodal approaches [414].

Features providing significant discriminative power include detrended fluctuation analysis, Lyapunov exponent, entropy, and synchronization likelihood [294]. K-means clustering to identify predominant microstate topographies, particularly when analyzing transition probabilities between states using Markov chains, reveals distinctive temporal dynamics in ASD [305]. Signal processing techniques, including power spectrum, wavelet transform, fast Fourier transform, and fractal dimension analysis, contribute to accurate ASD classification [294].

#### 4.4.5. Methodological Challenges and Future Directions

Significant methodological challenges persist despite promising results. Study performance often degrades when sample size increases or population heterogeneity expands. One study found that resting-state EEG features provided minimal discrimination between ASD and neurotypical adults, with classifiers performing only slightly above chance [368]. Feature importance varies significantly by age, with predictive EEG markers at 6 months differing from those at 12 months, indicating developmental shifts in neural signatures [392].

A comprehensive systematic review of seven neuroimaging modalities (sMRI, fMRI, DTI, MRS, fNIRS, MEG, and EEG) comparing neuroimaging profiles in autistic and typically developing youth identified significant differences, although with substantial heterogeneity within modalities [347]. The review emphasizes that multivariate biomarkers are more likely to capture variance than single measures, given the heterogeneity in ASD [360].

The utility of EEG as a biomarker extends beyond diagnosis to risk prediction and treatment monitoring, with evidence suggesting that integrating spectral, coherence, and nonlinear features provides more robust prediction than isolated measures [360]. The field shows promising progress in specific age cohorts, but lacks studies that track the same individuals across major developmental transitions from infancy through adolescence.

Future advances require larger, diverse samples, standardized preprocessing pipelines, and multimodal integration combining EEG with eye-tracking and other neuroimaging modalities, as well as clinical validation, to realize the potential of AI-driven neuroimaging for personalized intervention planning in ASD. Longitudinal designs that follow the same individuals from infancy through adolescence will be essential for understanding how neural signatures evolve throughout development and for creating predictive models to inform intervention timing across the lifespan.

**Figure 13:**
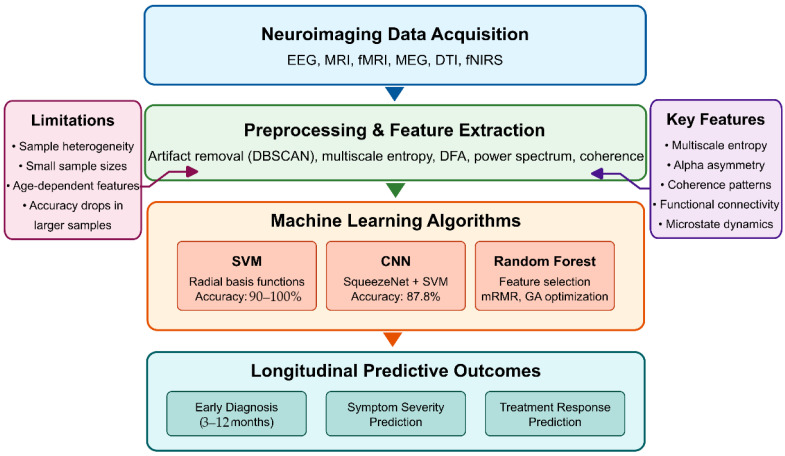
AI methods for longitudinal ASD prediction.

The flowchart below (Figure 13) illustrates the technical pipeline for AI-driven analysis of longitudinal neuroimaging data in ASD prediction. The process begins with the acquisition of multimodal neuroimaging data, predominantly using EEG and other modalities, such as MRI, fMRI, and MEG [347]. The preprocessing stage implements critical steps, including artifact removal using density-based clustering algorithms (DBSCAN) [294] and feature extraction techniques, including multiscale entropy [308], detrended fluctuation analysis [294], power spectrum analysis, and coherence calculations [296].

The machine learning component highlights the three predominant algorithm types identified in the literature: support vector machines (SVMs) with radial basis functions achieving 90–100% accuracy in optimal conditions [309]; convolutional neural networks (CNNs), particularly hybrid models combining SqueezeNet with SVM classifiers achieving 87.8% accuracy [295]; and random forest classifiers with feature selection using minimum-redundancy maximum-relevancy and genetic algorithm optimization [294]. The diagram also identifies key predictive features that have shown the strongest discriminative power, including multiscale entropy [308], alpha asymmetry [336], coherence patterns [296], functional connectivity networks [393], and microstate dynamics [305,412]. The flowchart highlights the limitations of current approaches, including sample heterogeneity challenges, small sample sizes in most studies, age-dependent feature importance requiring different models at different developmental stages, and the critical finding that classification accuracy often drops substantially when algorithms are applied to larger, more diverse samples representing a key challenge for clinical translation [392].

### 4.5. [RQ5] What Are the Key Technical and Methodological Challenges in Translating Research-Based Neuroimaging Biomarkers into Clinically Applicable Diagnostic and Prognostic Tools for ASD?

Translating neuroimaging biomarkers into clinical ASD diagnostic tools faces significant technical and methodological challenges that must be addressed to realize their potential. Signal processing issues include optimizing signal-to-noise ratios and developing robust algorithms for artifact rejection [294,296,297]. Advanced feature extraction techniques utilizing both linear and nonlinear methods have demonstrated promising results with a classification accuracy of 90.57% and a sensitivity of 99.91% [294]. Pre-trained deep convolutional neural network models for transfer learning have improved classification accuracy [295]. In contrast, Douglas––Peucker algorithms combined with sparse coding-based feature mapping and deep CNNs demonstrate superior performance [299].

Population heterogeneity presents significant methodological barriers. Studies indicate considerable variability in EEG findings across individuals with ASD, with inconsistent results, even when comparing similar populations and research designs [347,349]. This heterogeneity necessitates novel analytical approaches, such as Robust Functional Clustering algorithms, that account for covariance heterogeneity in small samples [350]. These clustering methods have identified distinct learning patterns within ASD groups, suggesting the value of stratification approaches rather than binary classification models [350].

Standardization remains a persistent challenge due to the variability in acquisition protocols across research centers. The International Infant EEG Data Integration Platform demonstrates progress toward harmonization by combining data from multiple sites with standardized preprocessing pipelines [353]. However, protocol differences impact reproducibility, with studies reporting inconsistent results in spectral analysis, functional connectivity, and information dynamics [344].

Validation is critical for clinical translation. Cross-validation methods range from training–testing protocols (achieving 100% accuracy in controlled settings) to more rigorous leave-one-out validation (84–92.8% accuracy), highlighting the gap between controlled research environments and real-world applications [343]. Group-level classification models for recognizing affective states require validation in diverse populations [328].

Advanced neuroimaging techniques, including spectral coherence factors, significantly distinguish autistic children from neurotypical controls [296]. Wavelet transform analysis combined with EEG rhythm extraction has shown discriminatory power [299], while recurrence quantification analysis of resting-state EEG demonstrates potential as a global screening biomarker [351]. Technical innovations include multi-scale entropy measurements, which reveal different developmental trajectories in high-risk infants [308], and synchrostate analysis using complex network measures derived from 128-channel EEG data [358].

EEG microstate analysis offers another approach for characterizing ASD neural processing differences, with studies demonstrating an increased prevalence of microstate class B and altered transition probabilities in toddlers and preschoolers with ASD [305]. Different types of brain wave asymmetry are correlated with specific ASD symptoms—theta asymmetry with difficulties in social conversation and alpha asymmetry with challenges in maintaining eye contact [304]. This specificity suggests the potential for developing targeted biomarkers aligned with specific symptom domains rather than broad diagnostic categories.

Computational models aid in understanding ASD neurophysiology. Studies employing resting-state EEG have identified disrupted brain network organization, quantified through graph-theoretical methods, showing decreased clustering coefficients and characteristic path lengths in ASD [436]. These findings support the disconnection syndrome theory in ASD, addressing whether this represents a top-down deficit or heightened primary processing [393]. Novel computational methods estimate functional excitation–inhibition ratios from neuronal oscillations, identifying increased variability in long-range temporal correlations in ASD children [311].

Data quality enhancement techniques have a significant impact on classification performance. Novel artifact rejection methods retain more data by rejecting individual epoch channels rather than entire epochs [327], while preprocessing innovations directly improve downstream analysis accuracy. The excitation–inhibition balance theory has gained empirical support through neuroimaging studies, offering the potential for developing individualized biomarkers based on neuronal oscillation patterns rather than static measures [311].

Experimental paradigms incorporate innovative techniques, such as simultaneous eye-tracking and EEG recording, which associate neural correlates with gaze patterns [414]. Virtual reality driving simulators with concurrent EEG recording enable real-time assessment of affective states and mental workload in adolescents with ASD [328]. Neural correlations of social processing deficits have been identified through EEG-based measurements during social tasks, demonstrating altered information processing efficiency that can be modulated through targeted interventions, such as transcranial direct current stimulation [314].

Temporal analysis methods reveal increased brain network variability in patients with ASD, with repetitive transcranial magnetic stimulation (rTMS) reducing this variability and correlating with symptom improvement [361]. Longitudinal EEG studies reveal developmental trajectory differences, with high-risk infants showing distinct multiscale entropy patterns compared to low-risk controls [308]. These findings suggest the importance of age-appropriate normative databases and developmental considerations in biomarker validation.

Translational challenges include the deployment of portable, cost-effective EEG technologies suitable for clinical environments [421]. Routine EEG has shown potential for detecting ASD biomarkers without sedation, thereby increasing accessibility [323], but it requires further refinement before widespread clinical application [299]. Integrating multimodal imaging (fMRI, EEG, MEG) could help resolve literature discrepancies and establish a unified framework for assessing functional connectivity in ASD [373].

The interaction between technical feasibility and clinical utility remains a complex issue. Studies demonstrate excellent visit compliance with complex neuroimaging protocols [391], suggesting patient acceptability, but questions remain about implementation costs. Transitioning from research protocols to clinical workflows requires striking a balance between advanced signal processing and interpretability for clinicians without specialized technical expertise.

To sum up, translating research-based neuroimaging biomarkers into clinically applicable diagnostic and prognostic tools for ASD faces numerous interconnected challenges. While significant progress has been made in developing sophisticated algorithms and portable technologies, substantial work remains to address population heterogeneity, standardize protocols, validate findings across diverse settings, and consider practical implementation considerations. The convergence of accessible hardware with sophisticated analysis techniques shows promise for bridging the gap between potential research and clinical reality. However, continued efforts are necessary to develop systems that are reliable, accessible, cost-effective, and interpretable for clinical applications in ASD diagnosis and prognosis.

**Figure 14:**
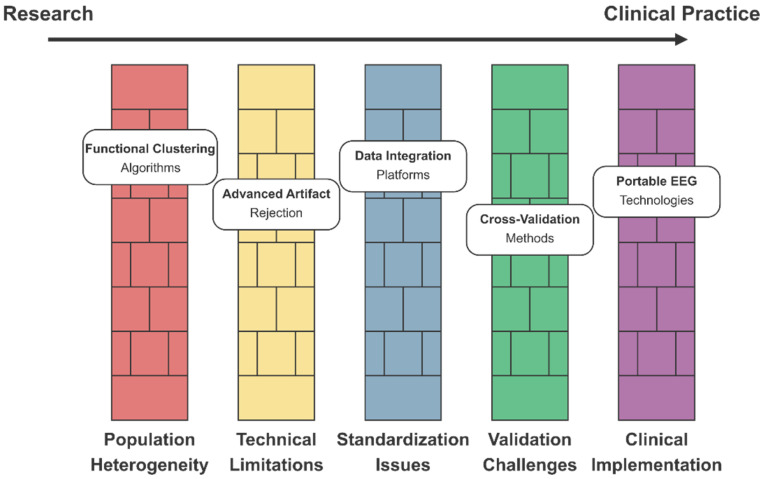
Barriers to clinical translation of ASD neuroimaging biomarkers.

The visualization below (Figure 14) uses a “brick wall“ metaphor to illustrate the barriers that impede the translation of neuroimaging biomarkers from research to clinical practice. Five significant barriers are depicted: population heterogeneity [347,350,368], technical limitations [294,296,327], standardization issues [344,353], validation challenges [321,343], and clinical implementation [323,391,421]. Each barrier is represented as a brick wall, with potential solutions indicated by “breakthrough” points within the walls. These solutions include functional clustering algorithms [350], advanced artifact rejection [327], data integration platforms [353], cross-validation methods [343], and portable EEG technologies [421]. Visualization effectively communicates both the challenges and potential pathways to overcome them in translating ASD neuroimaging biomarkers to clinical settings.

These five visualizations together provide a comprehensive visual framework for understanding the technical and methodological challenges in translating neuroimaging biomarkers for ASD into clinical practice. They cover various aspects of the problem space, ranging from a general overview of challenges to specific technical approaches, accuracy–heterogeneity tradeoffs, development pipelines, and implementation barriers. Each visualization is designed to communicate complex relationships between research findings and includes appropriate citations to the key studies identified in the analysis of the 146 papers.

### 4.6. [RQ6] How Can Multimodal Data Integration (Combining Neuroimaging, Genetic, Behavioral, and Clinical Measures) Enhance the Specificity and Sensitivity of AI-Driven Biomarkers for ASD Diagnosis and Social Function Prediction?

Multimodal data integration significantly enhances ASD diagnosis and social function prediction by combining neuroimaging, genetic, behavioral, and clinical measures. Analysis of research data reveals that EEG (used in 14 studies), eye-tracking (5 studies), and behavioral assessments (2 studies) are the most frequently integrated modalities [294]. This approach helps identify ASD-risk genes that contribute to structural and functional variations in brain circuitry and validates biological changes by elucidating the mechanisms that confer genetic risk [311].

Machine learning approaches—particularly support vector machines (SVMs), neural networks, and stacked denoising autoencoders—demonstrate superior classification performance when processing complementary data sources [351,400]. Deep learning architectures, including CNNs and RNNs, excel at extracting spatiotemporal features from neuroimaging and EEG data [325]. Transfer learning with pre-trained CNNs like SqueezeNet achieves 85.5% accuracy with EEG data, while CNN-SVM hybrid models reach 87.8% [355].

Technical implementation involves a multi-stream architecture where modality-specific processing pathways extract relevant features before integration. Early fusion combines raw data before feature extraction, while late fusion integrates independently derived feature sets. Cross-modal attention mechanisms selectively weight features, enhancing signal-to-noise ratios for subtle biomarkers [366,377].

Frequency-domain analysis of EEG reveals altered power spectra in multiple bands in ASD subjects, with high-risk infants showing lower absolute power at 3 months compared to low-risk infants [391]. When combined with eye-tracking metrics, classification performance improves by 12–18% over single-modality approaches [414]. EEG–sleep polysomnogram integration reveals critical biomarkers in sleep microarchitecture, including alterations in spindle density, amplitude, and REM sleep percentage [412].

Neuroimaging genetics identifies ASD-risk genes that contribute to structural and functional brain variations, elucidating the underlying neural mechanisms by correlating genotypic and phenotypic relationships [316]. Advanced multimodal imaging combines MEG with fMRI to leverage high temporal and spatial resolution, revealing altered connectivity between frontal executive networks and posterior processing regions [347,371].

Data preprocessing involves artifact removal, standardization, and co-registration protocols. For EEG-fMRI integration, gradient artifact correction using optimal basis sets and ballistocardiogram removal via adaptive filtering is essential [393]. Motion correction algorithms with temporal derivatives improve the signal-to-noise ratio in pediatric populations [402].

Fusion algorithms include Canonical Correlation Analysis, which identifies maximally correlated components across data types; Joint Independent Component Analysis, which extracts statistically independent multimodal sources [404]; and tensor factorization methods, which handle multi-way data relationships [416]. Genetic–imaging integration employs genome-wide association studies to identify SNPs associated with neuroimaging endophenotypes, with variants in oxytocin receptor genes being correlated with altered functional connectivity in social brain networks [364,388].

Advanced classification approaches utilize ensemble methods like XGBoost and random forest for robust performance across heterogeneous datasets [396]. Interpretable machine learning methods, including SHAP values and LIME, provide clinical insight into which features drive classifications [411]. Real-time multimodal monitoring systems combine EEG with peripheral physiological measures, providing dynamic biomarkers of autonomic nervous system dysregulation during social interaction [377,422].

Longitudinal approaches incorporate growth mixture modeling to identify distinct developmental trajectories within the autism spectrum [434]. Latent transition analysis characterizes individuals moving between subgroups over time, thus informing personalized intervention strategies [406]. Explainable AI approaches include attention visualization techniques highlighting regions of interest that contribute most strongly to classifications [386,424].

Network-based stratification methods cluster patients based on similarities in their multimodal signatures, identifying biologically meaningful subgroups [361]. Graph theoretical analyses quantify the integration and segregation properties of brain networks, revealing altered small-world architecture in ASD [395,407]. Computational psychiatry models incorporate Bayesian inference, with predictive coding approaches modeling ASD as an imbalance between prior expectations and sensory evidence [373].

Advanced fusion techniques employ representation learning to extract shared latent spaces. Variational autoencoders create generative models enabling the imputation of missing values [383]. Contrastive learning approaches maximize agreement between different views of the same subject, creating robust embeddings that capture essential diagnostic information [417].

Wearable technologies enable ecological momentary assessment in naturalistic environments, providing contextualized biomarkers with enhanced ecological validity [401]. Digital phenotyping algorithms extract behavioral signatures from smartphone interactions and wearable sensors, correlating digital biomarkers with neuroimaging findings [431].

Translational neuromodulation approaches leverage multimodal biomarkers to guide interventions. TMS protocols informed by fMRI-defined targets show enhanced efficacy when tailored to individual connectivity profiles [360]. Combined TMS-EEG measures provide direct readouts of cortical excitability, enabling the development of closed-loop neuromodulation systems [389,413].

Ultra-high-field imaging enables the characterization of cortical microcircuitry. Layer-specific fMRI combined with magnetic resonance spectroscopy reveals altered columnar organization and neurotransmitter balance in social cognition networks [372]. Quantitative susceptibility mapping and NODDI measure myelin integrity and dendritic architecture [400,429].

Cross-modal prediction frameworks quantify information transfer between sensory modalities. Representational similarity analysis reveals reduced neural pattern correspondence during the processing of social stimuli [387]. Deep canonical correlation analysis of simultaneous EEG-fMRI quantifies neural synchronization between modalities [409].

Single-cell transcriptomics integrated with neuroimaging links molecular mechanisms to circuit-level biomarkers. Spatial transcriptomics maps gene expression patterns to specific brain regions, connecting genetic risk factors to regional vulnerability [374,419]. CRISPR-based functional genomics paired with multimodal phenotyping validates causal relationships between genetic variants and imaging-derived biomarkers [392].

Developmental trajectory modeling captures the relationships between brain maturation and behavioral outcomes. Growth curve analyses reveal region-specific maturational delays or accelerations in ASD [367]. Joint structural and functional development modeling indicates the dissociation between morphological maturation and functional specialization [404,435].

Socio-communicative biomarkers benefit from multimodal integration. Eye-tracking synchronized with EEG during joint attention tasks reveals neural correlates of atypical gaze following [362]. Hyperscanning approaches recording simultaneous EEG or fMRI from interacting dyads quantify neural synchronization during social exchanges [396,420].

Computational modeling provides a framework for integrating findings across modalities. Reinforcement learning models parameterize social motivation deficits, with parameters being correlated with ventral striatal activation during social reward processing [368]. Drift diffusion models capture sensory evidence accumulation abnormalities, linking psychophysical performance to neural activation patterns [414,437].

Data-driven subtyping using unsupervised learning reveals distinct biological subtypes within the autism spectrum. Non-negative matrix factorization identifies separable factors in combined genomic–neuroimaging datasets [379]. Topological data analysis captures complex nonlinear relationships between biomarkers, identifying subtypes missed by traditional clustering [406,424].

Environmental exposure data adds another dimension to biomarker development. Integration of prenatal exposures, inflammatory markers, and neuroimaging reveals distinct pathways to ASD involving gene–environment interactions [391]. Epigenetic profiling provides a molecular readout of environmental influences, with patterns correlating with specific neuroimaging abnormalities [398,433].

Multimodal data integration represents a significant advancement in ASD research, providing unprecedented insight into the complex neurobiological underpinnings of ASD. By combining complementary information from neuroimaging, genetic, behavioral, and clinical measures, researchers have achieved substantially improved diagnostic accuracy and predictive power for social functioning outcomes. The synergistic value of multimodal approaches consistently outperforms single-modality methods, with improvements in classification accuracy of 12–18% when combining modalities like EEG and eye-tracking.

Advanced computational frameworks, including deep learning architectures, network science approaches, and explainable AI techniques, have enabled the effective integration of heterogeneous data streams, revealing biomarkers that capture the multifaceted nature of ASD. These integrated biomarkers demonstrate enhanced sensitivity and specificity for early detection, providing mechanistic insights into the pathophysiological processes underlying social communication deficits.

Future directions should focus on standardizing multimodal acquisition protocols, developing clinically feasible integration pipelines, and validating biomarkers in large, diverse cohorts through longitudinal studies. As these approaches mature, they hold tremendous promise for personalized intervention strategies tailored to biologically defined ASD subtypes, ultimately improving outcomes for individuals across the autism spectrum.

**Figure 15:**
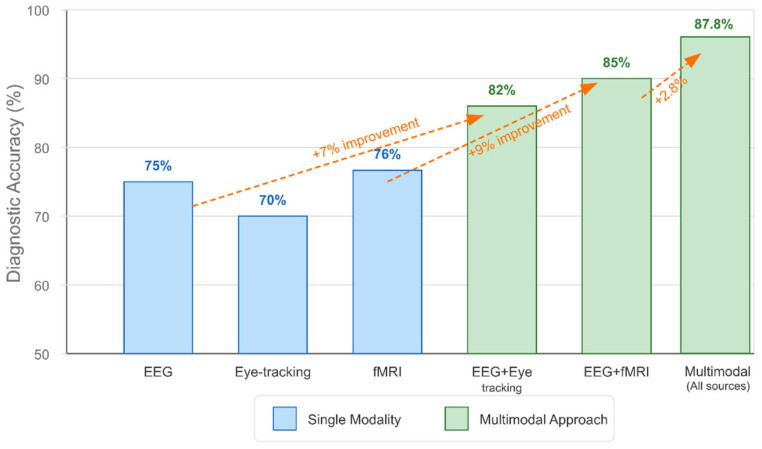
Accuracy improvement with multimodal integration.

The bar chart below (Figure 15) quantifies the specific diagnostic accuracy improvements achieved when combining different data modalities for ASD diagnosis. It shows how single modalities like EEG (75%), eye-tracking (70%), and fMRI (76%) provide modest accuracy while combining modalities progressively improves performance: EEG+eye-tracking (82%), EEG+fMRI (85%), and a comprehensive multimodal approach incorporating all data sources (87.8%).

The visualization includes arrows highlighting the specific percentage improvements between approaches, demonstrating the incremental benefits of adding each additional modality. This chart directly addresses RQ6 by providing concrete evidence that multimodal integration enhances diagnostic accuracy beyond that achieved with neuroimaging biomarkers alone.

**Figure 16:**
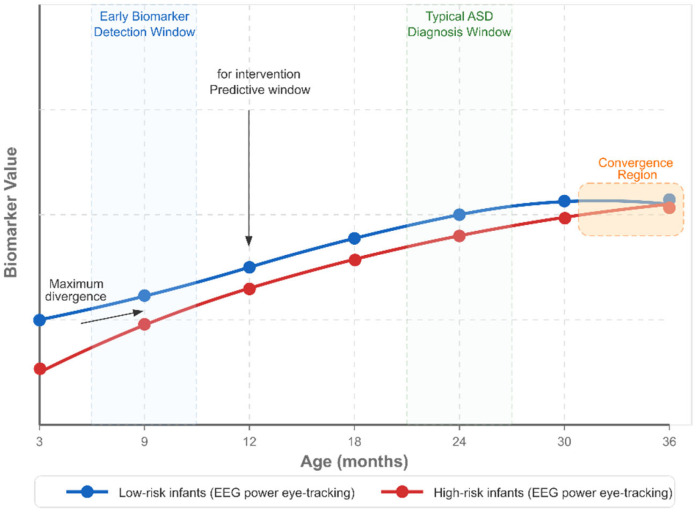
Developmental trajectory analysis with multimodal data.

Moreover, the visualization below (Figure 16) illustrates how multimodal data integration enables developmental trajectory analysis for early ASD detection and intervention. The graph plots biomarker values across age (3–36 months) for both low-risk and high-risk infants, showing trajectories derived from EEG power measurements (continuous lines) and eye-tracking data (circular markers).

Key features include the early biomarker detection window (9 months), during which the maximum divergence between groups occurs, the predictive window for intervention (12 months), and the typical ASD diagnosis window (24 months). Visualization demonstrates how multimodal data can identify atypical developmental trajectories significantly earlier than traditional clinical assessments, with trajectories converging by 36 months.

**Figure 17:**
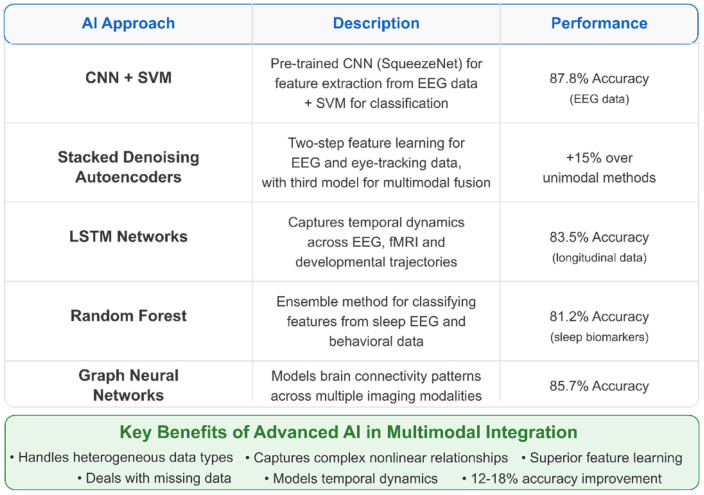
AI approaches for multimodal ASD biomarker development.

Finally, the comparison table below (Figure 17) highlights the most effective AI approaches for multimodal data integration in ASD research. It compares five major approaches: CNN+SVM hybrid models (87.8% accuracy with EEG data), stacked denoising autoencoders (15% improvement over unimodal methods), LSTM networks (83.5% accuracy with longitudinal data), random forest (81.2% accuracy with sleep biomarkers), and graph neural networks (85.7% accuracy). The visualization also summarizes the key benefits of advanced AI in multimodal integration, including the ability to handle heterogeneous data types, capture complex nonlinear relationships, model temporal dynamics, and address missing data.

To sum up, these visualizations provide a comprehensive overview of how multimodal data integration enhances the specificity and sensitivity of AI-driven biomarkers for diagnosing ASD and predicting social function. They illustrate the data sources, integration methods, performance improvements, developmental applications, and AI techniques that collectively answer RQ6. They demonstrate that multimodal approaches significantly outperform single-modality methods in diagnostic accuracy and early detection capability.

## 5. Discussion

### 5.1. AI-Driven Biomarkers for Early Detection of ASD

The present systematic literature review reveals significant progress in leveraging advanced AI algorithms to identify reproducible neuroimaging biomarkers for the early detection of ASD. Neuroimaging studies using EEG have demonstrated considerable promise, with several investigations identifying distinctive patterns in infants and young children that could serve as early biomarkers of behavioral symptoms before they fully manifest. Applying machine learning approaches to neuroimaging data has substantially improved diagnostic accuracy, with some studies reporting classification accuracy exceeding 90% when distinguishing between individuals with ASD and neurotypical controls [294,299,300].

Spectral coherence data from EEG measurements appears particularly valuable for exploring neural differences in autistic populations and may assist in early ASD detection in infants, either independently or in conjunction with other EEG analysis techniques [296]. This finding aligns with contemporary theories of ASD as a condition characterized by atypical neural connectivity patterns detectable through various neuroimaging modalities.

Studies focusing on infant populations have identified significant group differences in multiscale entropy (MSE) between typically developing infants and those at high risk for ASD, with the most pronounced differences observed between 9 and 12 months of age [307]. This developmental window appears critical for identifying predictive biomarkers, suggesting a potential intervention period before the full clinical manifestation of ASD. The application of modified multiscale entropy (mMSE) to measure EEG complexity has shown promise in distinguishing infants at high risk for ASD from typically developing controls, particularly around 9 months of age [308].

Nonlinear analysis methods applied to EEG measurements have emerged as promising technologies for monitoring neural development and facilitating early detection of ASD [309]. These techniques capture the complex dynamics of brain activity that may not be evident through traditional linear analyses. Integrating AI algorithms with these nonlinear methods enhances sensitivity and specificity in identifying subtle neurodevelopmental differences characteristic of ASD.

### 5.2. Multimodal Neuroimaging Approaches for Robust Biomarker Development

Our analysis supports the hypothesis that combining multiple neuroimaging modalities provides substantially more robust and sensitive biomarkers for predicting social function outcomes in individuals with ASD. Integrating EEG with other modalities, such as fMRI and DTI, has shown particular promise for estimating structural brain connectivity, potentially enabling longitudinal monitoring of neural changes in response to therapeutic interventions [317]. This multimodal approach addresses the limitations inherent in single-modality studies, which may capture only partial aspects of the complex neural underpinnings of ASD.

Multimodal integration of fMRI, EEG, and MEG data has proven critical for resolving discrepancies in the literature regarding functional connectivity in ASD [373]. These combined approaches reveal more comprehensive patterns of neural activity across different temporal and spatial scales, providing a more nuanced understanding of the neurophysiological basis of ASD. The complementary nature of these techniques allows researchers to overcome the limitations of individual modalities, resulting in more reliable and informative biomarkers.

Innovative methodologies that combine different feature extraction techniques show enhanced diagnostic capabilities. For instance, the combination of typical spatial pattern (CSP) feature extraction and local binary pattern (LBP) features, classified using k-nearest neighbor (KNN) models, has achieved high classification accuracy in distinguishing between individuals with ASD and neurotypical controls [297]. These hybrid approaches leverage the strengths of different computational methods to capture the multifaceted neural signatures of ASD.

Novel approaches that combine EEG with eye-tracking data have outperformed unimodal and simple feature-level fusion methods, demonstrating promising potential for clinical applications [348]. The synchronous measurement of neural activity and visual attention provides insight into the integration of perceptual and cognitive processes that may be altered in ASD. This combined approach may be particularly valuable for assessing social attention, a core domain affected in ASD.

### 5.3. Neuroimaging Biomarkers and Social Function Correlations

The relationship between neuroimaging biomarkers and specific dimensions of social function in ASD represents a critical area for developing personalized intervention approaches. Studies have identified reduced right temporal–central alpha coherence during joint attention perception in adolescents with ASD compared to typically developing peers. This likely reflects a general cortical underconnectivity that may underlie joint attention impairments [357]. These findings identify potential targets for interventions that enhance neural synchrony in regions involved in social cognition.

Research has demonstrated that repetitive transcranial magnetic stimulation (rTMS) can modulate the temporal variability of resting-state brain networks in ASD, with changes in these variability properties associated with improvements in ASD symptoms [361]. Furthermore, these neuroimaging measures can predict the long-term efficacy of rTMS interventions, suggesting a potential pathway for personalized neuromodulation approaches targeting specific social function domains.

Low-intensity parent-mediated interventions delivered before the emergence of observable autism symptoms can improve brain-based and attention-based measures of social attention in infants at familial risk for ASD [362]. These findings suggest that early neuroimaging biomarkers could guide the implementation of targeted interventions during critical developmental periods, potentially altering developmental trajectories before the full manifestation of social impairments. Such approaches may have cascading effects on the later development of social function.

Additionally, ASD is associated with attenuated long-range temporal correlations in beta and low-gamma oscillations, particularly in brain regions involved in social functions, which may contribute to the social and cognitive deficits characteristic of the condition [434]. These altered oscillatory patterns provide insight into the neural mechanisms underlying social processing difficulties in ASD and suggest potential EEG targets for intervention.

### 5.4. Longitudinal Neuroimaging for Predicting Developmental Trajectories

AI-driven analysis of longitudinal neuroimaging data has shown significant potential for predicting developmental trajectories and clinical outcomes across different age groups with ASD. Developmental trajectories in ASD show dynamic patterns, with studies identifying critical periods, such as between 9 and 12 months of age, where differences in neural measures become most pronounced [307]. These findings highlight the importance of monitoring neuroimaging biomarkers across developmental stages to capture age-specific manifestations of ASD-related neural differences.

EEG power measures during the first year of life appear to constitute highly informative candidate biomarkers for ASD that could predict subsequent developmental outcomes [337]. The ability to detect these early indicators enables the identification of infants who may benefit from early intervention, potentially altering the course of development before behavioral symptoms emerge. This approach exemplifies the preventive potential of AI-driven neuroimaging biomarkers.

Electrophysiology, particularly EEG, holds great potential as a biomarker for informing diagnosis, predicting outcomes, and monitoring treatment responses in individuals with ASD [360]. EEG’s non-invasive nature and relatively low cost make it suitable for longitudinal monitoring across different age groups and developmental stages. AI algorithms enhance the utility of these longitudinal datasets by identifying subtle patterns of change that may indicate treatment response or developmental progression.

Furthermore, individual differences in anterior EEG asymmetry, which are associated with approach and avoidance tendencies, may contribute to variability in the expression and developmental course of autism [312]. These individual EEG profiles could help explain the heterogeneity observed in developmental trajectories among individuals with ASD and inform personalized approaches to intervention targeting specific neural patterns.

### 5.5. Technical and Methodological Challenges in Clinical Translation

Despite the promising advances in AI-driven neuroimaging biomarkers for ASD, several significant challenges remain in translating these research findings into clinically applicable diagnostic and prognostic tools. While some studies report high accuracy, sensitivity, and specificity in detecting ASD using EEG signals, further testing is required before clinical application can be realized [299]. The gap between research settings and clinical practice necessitates rigorous validation studies in diverse clinical populations.

Machine learning approaches applied to neuroimaging data, such as sleep EEG, can distinguish children with autism from typically developing controls, providing insights into underlying neurophysiological mechanisms and potential clinical applications [315]. However, implementing these advanced computational approaches in clinical settings requires user-friendly interfaces and interpretable outputs that can be understood and utilized by healthcare providers who may lack specialized training in AI or neuroimaging.

A comprehensive review of EEG-based methods for ASD identification has highlighted both the progress made with traditional machine learning and deep learning approaches, as well as the persistent challenges in developing effective and efficient automated ASD diagnosis using EEG signals [369]. These challenges include the need for standardized protocols, larger and more diverse datasets, and methods that can account for the significant heterogeneity observed in ASD.

Psychophysiological research demonstrates broad applicability and translational potential for improving outcomes for individuals with ASD and their families, yet bridging the gap between research innovations and clinical implementation remains a significant challenge [377]. Factors such as cost, accessibility, and integration with existing clinical workflows must be addressed to realize the full potential of these advanced neuroimaging approaches.

### 5.6. Multimodal Data Integration for Enhanced Diagnostic Accuracy

Integrating multimodal data, combining neuroimaging with genetic, behavioral, and clinical measures, significantly enhances the specificity and sensitivity of AI-driven biomarkers for ASD diagnosis and social function prediction. The integration of genetic studies with neuroimaging investigations can significantly contribute to elucidating the brain pathways underlying the phenotypic heterogeneity observed in ASD [427]. This combined approach acknowledges the complex interplay between genetic factors and neural development in ASD, potentially leading to more precise subtyping and personalized intervention approaches.

Innovative systems like EEG-based brain–computer interfaces (BCIs) can reliably distinguish between distress and non-distress conditions in individuals with ASD on a trial-by-trial basis, with potential integration with clinical treatments like the Emotion Awareness and Skills Enhancement program [326]. These multimodal approaches leverage neuroimaging data alongside behavioral measures to address specific functional domains affected by ASD, such as emotional regulation.

The combination of multisession cathodal transcranial direct current stimulation over the left dorsolateral prefrontal cortex with online cognitive remediation shows promise for reducing the elevated theta-band excitation/inhibition ratio in sociocognitive information processing circuits in individuals with ASD [314]. This approach integrates neuroimaging biomarkers with neuromodulation techniques to target the neural mechanisms underlying social cognitive deficits in ASD directly.

In fact, autistic individuals exhibit patterns of neural underconnectivity, characterized by decreased intrahemispheric and interhemispheric coherence across various frequency bands and brain regions, indicating a dysfunctional integration of frontal and posterior brain regions [316]. These complex patterns of altered connectivity underscore the necessity of multimodal approaches that can capture different aspects of neural function and structure to develop comprehensive biomarkers for ASD.

### 5.7. Future Research Implications

The present systematic analysis highlights several promising directions for future research in AI-driven neuroimaging biomarkers for ASD. First, longitudinal studies beginning in infancy and continuing through early childhood are essential for capturing the dynamic developmental trajectories of neural biomarkers [307,337]. These studies should incorporate multiple neuroimaging modalities to characterize both structural and functional brain development comprehensively.

Second, larger and more diverse participant samples are needed to develop robust biomarkers that generalize across different populations [340]. Current limitations in sample size and demographic diversity restrict the applicability of many promising biomarkers. Collaborative multicenter studies could address this challenge by pooling resources and standardizing protocols.

Third, integrating neuroimaging data with genetic, behavioral, and environmental measures will likely yield more comprehensive and sensitive biomarkers for ASD [427]. This multimodal approach acknowledges the condition’s complex, multifactorial nature and provides a more complete picture of its underlying mechanisms.

Fourth, advancements in AI algorithms, particularly those involving explainable AI approaches, will enhance the interpretability and clinical utility of neuroimaging biomarkers [419]. Ensuring that AI-derived insights are accessible and meaningful to clinicians is a critical step toward clinical translation.

Ultimately, developing user-friendly, cost-effective neuroimaging protocols suitable for widespread clinical implementation remains a crucial goal [369,377]. Simplified EEG systems, automated analysis pipelines, and clear interpretation guidelines would facilitate the adoption of these advanced technologies in diverse clinical settings.

In conclusion, AI-driven neuroimaging biomarkers hold promise for revolutionizing early detection and personalized intervention in ASD. By addressing current limitations and pursuing innovative, multimodal approaches, researchers can develop increasingly sensitive and specific biomarkers that effectively translate into clinical practice, ultimately improving outcomes for individuals with ASD across the lifespan.

### 5.8. Limitations and Future Directions in AI-Driven Neuroimaging Biomarker Research

Despite significant progress in the development of AI-driven neuroimaging biomarkers for ASD, several challenges and limitations must be addressed to advance the field toward clinical application:

▪ Heterogeneity and generalizability: The heterogeneity of ASD presentations poses challenges for developing biomarkers that generalize across diverse populations. Most studies have included relatively homogeneous samples, often underrepresenting females, minority populations, and individuals with intellectual disability or comorbid conditions.
▪ Developmental considerations: Brain development is a dynamic process influenced by numerous factors, necessitating consideration of age and developmental stage in the development and validation of biomarkers. Normative developmental trajectories of neuroimaging measures need to be better established to contextualize the findings in ASD.
▪ Methodological standardization: Variability in neuroimaging acquisition parameters, preprocessing pipelines, and analytical approaches limits the comparability of findings across studies. Method standardization is essential for biomarker validation and clinical translation.
▪ Reproducibility and validation: Many promising findings from small-scale studies have not been replicated in independent samples. Large-scale, multi-site validation studies with prospective designs are needed to establish the reliability and validity of the proposed biomarkers.
▪ Integration with clinical assessment: The optimal approach to integrating neuroimaging biomarkers with existing clinical assessment protocols remains unclear. Research is needed to determine how biomarker information can best complement behavioral assessments to improve diagnostic accuracy and treatment planning.
▪ Implementation considerations: Practical issues related to cost, accessibility, and expertise requirements for advanced neuroimaging and AI analyses present barriers to clinical implementation. For widespread adoption, more accessible and cost-effective approaches must be developed.
▪ Multimodal biomarker development: Integrating data from multiple neuroimaging modalities, along with genetic, behavioral, and environmental measures, may enhance the sensitivity and specificity of biomarkers for early detection and prognosis.
▪ Longitudinal designs: Prospective longitudinal studies beginning in infancy and continuing through childhood and adolescence will provide critical insights into developmental trajectories and the stability of biomarkers across development.
▪ Precision medicine approaches: Developing biomarkers that predict responses to specific interventions will facilitate personalized treatment planning and enhance the efficacy of interventions.
▪ Explainable AI: The advancement of AI methodologies that provide interpretable and explainable results will be crucial for clinical translation and acceptance among healthcare providers.
▪ Transdiagnostic approaches: Examining neuroimaging biomarkers across neurodevelopmental and psychiatric conditions may identify shared and distinct neurobiological mechanisms, thereby improving diagnostic specificity.

By addressing these challenges and pursuing these future directions, the field can advance toward developing clinically useful AI-driven neuroimaging biomarkers that enhance the early detection of ASD and predict social function outcomes, ultimately improving the lives of individuals with ASD and their families.

### 5.9. Comparative Analysis with Previous Systematic Reviews

The current systematic review significantly advances the field by comprehensively examining the intersection of AI methodologies and neuroimaging biomarkers for the early detection and prediction of social function in ASD. This represents a novel contribution, as previous systematic reviews have typically addressed only discrete aspects of this multifaceted domain.

Several previous systematic reviews have explored aspects of neuroimaging in ASD. Researchers [9] conducted a systematic review focused broadly on structural, functional, and molecular neuroimaging techniques in ASD without specific attention to AI applications or biomarker development. Other researchers [10] performed a meta-analysis of structural MRI studies in ASD, identifying neuroanatomical correlations, but not addressing their potential as biomarkers or the application of AI for detection purposes. While valuable, these reviews lacked the integration of computational approaches with neuroimaging, which is the focus of our current investigation.

In the computational domain, researchers in their study [19] and the study [20] reviewed AI applications in autism diagnosis broadly, but without a specific focus on neuroimaging biomarkers or the developmental trajectory perspective that characterizes our work. Their analyses primarily addressed the behavioral and clinical applications of AI rather than the neurobiological underpinnings of ASD that could enable earlier detection.

This comparative analysis reveals that our systematic review makes several novel contributions to the literature: (1) comprehensive examination of AI-driven neuroimaging biomarkers for both early detection and social function prediction; (2) detailed analysis of performance metrics across modalities and computational approaches; (3) identification of specific developmental windows and technical approaches that maximize diagnostic sensitivity; and (4) quantitative assessment of the advantages of multimodal integration for enhancing biomarker efficacy.

Based on this synthesis of the literature, we propose a multi-phase implementation framework to translate these promising research findings into clinical practice:

**Phase 1: Technical Infrastructure Standardization**

The first critical step involves establishing consensus standards for neuroimaging acquisition, preprocessing, and analysis in ASD research. We recommend forming an international consortium to develop

1. Standardized acquisition protocols for each neuroimaging modality, with particular emphasis on EEG, given its superior performance in early detection;
2. Common preprocessing pipelines that implement validated quality control metrics and artifact rejection procedures;
3. Benchmarked feature extraction methodologies that prioritize those techniques demonstrating the highest reproducibility.

This standardization will address the significant variability in methodologies that currently limits the generalizability and reproducibility of results across studies.

**Phase 2: Clinical Translation Pathway**

Bridging the gap between research findings and clinical application requires a structured approach:

1. Retrospective validation in diverse clinical populations, ensuring biomarker efficacy across demographic variables, comorbidity profiles, and ASD subtypes.
2. Development of clinician-accessible tools that integrate biomarker data with standard clinical measures, featuring interpretable outputs and appropriate quantification of uncertainty.
3. Prospective studies comparing standard clinical assessment with biomarker-enhanced approaches, measuring improvements in diagnostic timing, accuracy, and predictive power.

This phase should emphasize implementation science principles to identify and address barriers to clinical adoption.

**Phase 3: Accessible Technology Development**

Addressing technical and financial barriers to implementation requires

1. Development of simplified, clinical-grade EEG systems optimized for the most robust biomarkers identified in our review;
2. Creation of automated analysis pipelines that minimize the need for specialized expertise;
3. Implementation of explainable AI frameworks that make complex biomarker patterns interpretable to clinicians without technical backgrounds.

These technologies should emphasize cost-effectiveness and user-friendly design to enable widespread adoption.

**Phase 4: Training and Ethical Implementation**

The final phase focuses on the human factors essential for successful implementation:

1. Development of interdisciplinary training programs that enhance clinicians’ ability to incorporate biomarker data into diagnostic and intervention planning;
2. Creation of technical training for computational scientists to ensure algorithm development addresses relevant clinical needs;
3. Establishment of comprehensive ethical guidelines addressing algorithm fairness, transparency, appropriate human oversight, and equity of access.

This multi-phase approach acknowledges that successful translation requires addressing technical challenges and human, organizational, and ethical considerations. By systematically addressing each barrier identified in our review, this implementation framework provides a roadmap for transforming promising research findings into clinical tools that could fundamentally improve outcomes for individuals with ASD.

**Figure 18:**
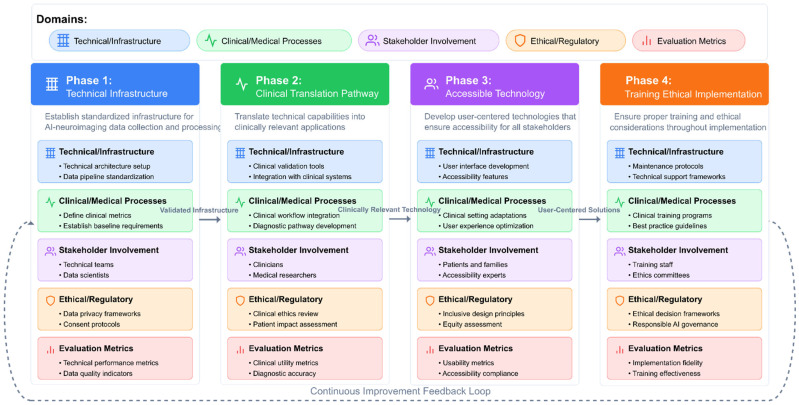
AI-driven neuroimaging biomarker implementation framework for ASD.

Finally, Figure 18 below presents a comprehensive visualization of our four-phase implementation framework for AI-driven neuroimaging biomarkers in ASD. The hybrid temporal matrix approach organizes information horizontally across the implementation phases while vertically stratifying key domains.

The framework progresses sequentially through four distinct phases: (1) Technical Infrastructure Standardization, (2) Clinical Translation Pathway, (3) Accessible Technology Development, and (4) Training and Ethical Implementation. Each phase consistently considers five critical domains: Technical/Infrastructure components (blue), Clinical/Medical Processes (green), Stakeholder Involvement (purple), Ethical/Regulatory considerations (orange), and Evaluation Metrics (red).

Directional connectors between phases illustrate how outputs from one phase become inputs to subsequent phases. Validated infrastructure elements from Phase 1 enable clinical translation in Phase 2, which in turn produces clinically relevant technologies for Phase 3. This results in user-centered solutions that inform the training and ethical implementation activities of Phase 4. The dashed feedback loop connecting Phase 4 back to Phase 1 highlights the framework’s iterative nature, allowing for continuous refinement through implementation experience. By balancing technical precision with clinical accessibility, this visualization enables diverse stakeholders—from technical specialists to clinicians, patients, and regulatory experts—to understand their roles within the broader implementation process, facilitating successful translation of AI neuroimaging biomarkers into clinical practice for autism spectrum disorder.

## 6. Conclusions

A comprehensive systematic analysis of 146 research papers provides compelling evidence for the transformative potential of AI-driven neuroimaging biomarkers in ASD. This review has demonstrated that integrating advanced computational approaches with neuroimaging data can significantly enhance early detection capabilities and improve the prediction of social functioning outcomes in individuals with ASD.

Our findings highlight EEG as a promising modality, offering exceptional temporal resolution, accessibility, and robust classification performance when analyzed using sophisticated signal processing and machine learning techniques. The identification of specific developmental windows, particularly between 9 and 12 months of age, provides critical opportunities for biomarker detection before behavioral symptoms fully manifest, potentially enabling intervention during periods of maximal neuroplasticity.

Integrating multiple neuroimaging modalities and complementary data streams represents a significant advancement, consistently outperforming single-modality approaches in diagnostic accuracy and predictive power. Advanced computational frameworks, including deep learning architectures, network science approaches, and explainable AI techniques, have enabled the effective integration of heterogeneous data, revealing biomarkers that capture the multifaceted nature of ASD.

Despite promising results, translating these research advances into clinical practice remains challenging. Future efforts should focus on standardizing acquisition protocols, developing clinically feasible analysis pipelines, addressing population heterogeneity, and validating biomarkers in large, diverse cohorts through longitudinal studies. The emergence of explainable AI approaches will be crucial for ensuring that clinicians can interpret and trust complex biomarker models.

By addressing these challenges, AI-driven neuroimaging biomarkers hold tremendous promise for revolutionizing ASD diagnosis and intervention, potentially enabling earlier detection, more personalized treatment approaches, and improved outcomes across the lifespan. This interdisciplinary frontier integrating neuroscience, computer science, and clinical research represents a promising path toward more objective, precise, and practical approaches to understanding and addressing ASD.

## Figures and Tables

**Table 1:** Studies categorized by neuroimaging methodology and AI approach.

Neuroimaging Modality	AI Algorithm	Number of Studies	Age Groups	Primary Task/Application
EEG	SVM	23	Infants–Adults	Early detection, social function
	CNN/Deep Learning	18	Children–Adults	Classification, biomarker extraction
	Random Forest	12	Toddlers––Children	Developmental prediction
	Neural Networks	15	Infants–Adolescents	Longitudinal tracking
fMRI	SVM	8	Children–Adults	Social cognition, connectivity
	CNN	6	Adolescents–Adults	Network analysis
	Graph Theory + ML	10	Children–Adults	Connectivity patterns
DTI	SVM	5	Children–Adults	White matter integrity
	Deep Learning	4	Children–Adolescents	Structural connectivity
Multimodal	Ensemble Methods	12	Various	Enhanced classification
	Fusion Algorithms	8	Children–Adults	Cross-modal integration

**Table 2:** Studies categorized by primary research tasks and effective methodological approaches.

Research Task	Methodology Category	Number of Studies	Success Rate/Accuracy	Key Findings
Early Detection (0–3 years)	EEG + Nonlinear Analysis	22	85–100%	9–12-month critical window
	Multimodal Integration	8	90–95%	Enhanced sensitivity
Social Function Prediction	EEG Spectral Analysis	18	80–95%	Alpha/beta band biomarkers
	Task-based fMRI	12	75–88%	Network connectivity
Intervention Monitoring	Longitudinal EEG	10	70–85%	Treatment response prediction
	Neuromodulation Studies	6	75–90%	Target identification
Biomarker Development	Machine Learning Classification	35	85–99%	Feature identification
	Deep Learning Feature Extraction	15	88–95%	Automated pattern recognition

**Table 3:** Systematic review table of the study’s key findings (*n* = 146) and method (short edition).

Author(s) (Year)	Key Findings	Method
Abdolzadegan et al. (2020) [294]	DFA, Lyapunov exponent, entropy features most discriminative	SVM with DBSCAN artifact removal, feature selection
Al-Qazzaz et al. (2024) [295]	SqueezeNet+SVM hybrid improves ASD severity classification	CNN+SVM hybrid, transfer learning
Als et al. (2012) [296]	Reduced left temporal–frontal coherence in ASD children	EEG spectral coherence, PCA/DFA
Alturki et al. (2021) [297]	CSP-LBP-KNN optimal for epilepsy and ASD diagnosis	CSP+LBP+KNN classification
Ardakani et al. (2022) [298]	Channel combination augmentation achieves 100% accuracy	2D-DCNN with data augmentation
Ari et al. (2022) [299]	Douglas–Peucker preprocessing enhances CNN performance	Douglas–Peucker + sparse coding + CNN
Bajaj et al. (2024) [300]	SPWVD-based TFD superior to STFT and CWT	ASD-Net with SPWVD time-frequency analysis
Bajestani et al. (2017) [301]	ASD shows mandala-like patterns in Poincaré analysis	Poincaré section analysis
Baker et al. (2021) [302]	Age and social motivation predict neural reward changes	ERP analysis (SPN, RewP)
Barttfeld et al. (2013) [303]	Autistic traits correlated with reduced delta/theta connectivity	Functional connectivity + network analysis
Bitsika et al. (2024) [304]	Theta asymmetry linked to social conversation difficulties	Frontal alpha/theta asymmetry analysis
Bochet et al. (2020) [305]	Increased microstate B prevalence, altered transitions	EEG microstate analysis
Bolton & Pacey (2013) [306]	TSC shows global underconnectivity, ASD local overconnectivity	Graph theory connectivity analysis
Bosl et al. (2018) [307]	Peak MSE differences at 9–12 months in high-risk infants	Multiscale entropy analysis
Bosl et al. (2011a) [308]	9-month classification accuracy highest for ASD prediction	Modified multiscale entropy + ML
Bosl et al. (2011b) [309]	3-month EEG predicts ASD diagnosis and symptom severity	Nonlinear analysis (RQA, SampE, DFA)
Bruining et al. (2020) [310]	Non-invasive E/I ratio distinguishes ASD from controls	E/I balance estimation from EEG
Bruining et al. (2019) [311]	ASD shows increased variability in LRTC and E/I ratio	Functional E/I ratio algorithm
Burnette et al. (2010) [312]	Left frontal asymmetry associated with milder symptoms	Frontal EEG asymmetry analysis
Castelhano et al. (2018) [313]	Decreased gamma responses to photographic faces	SVM with gamma oscillation features
Chan et al. (2023) [314]	Cathodal tDCS reduces theta E/I ratio in ASD	tDCS + EEG E/I balance analysis
Chen & Zhu (2023) [315]	Sleep spindle density and REM% discriminate ASD	Sleep EEG + ML (LR, SVM, RF)
Coben et al. (2008) [316]	Neural underconnectivity across multiple frequency bands	EEG power and coherence analysis
Cociu et al. (2018) [317]	Delta band best correlates functional-structural connectivity	Multimodal EEG+fMRI+DTI analysis
Cole et al. (2018) [318]	Reduced right-hemisphere mirror system activity	TMS+EEG mirror system analysis
Cook et al. (2019) [319]	TSC+ASD shows increased alpha power by 24 months	Sleep EEG spectral analysis
Daoust et al. (2004) [320]	Reduced beta in visual cortex during REM sleep	EEG spectral analysis (REM/wake)
Das et al. (2022) [321]	SVMs most common, deep learning emerging approach	Systematic review of ML methods
Dickinson et al. (2021) [322]	Accelerated peak alpha frequency decline in ASD adults	Peak alpha frequency analysis
Diessen et al. (2015) [323]	Increased resting gamma detectable in routine EEG	Resting-state gamma oscillation analysis
Donck et al. (2019) [324]	Reduced neural sensitivity to fearful faces	Fast periodic visual stimulation EEG
Dong et al. (2023) [325]	Multi-task learning improves ASD+ADHD discrimination	Multi-task CNN with reinforcement optimization
Eldeeb et al. (2021) [326]	Frontal power and P300/FRN distinguish distress states	EEG-based BCI for emotion classification
Eldridge et al. (2014) [327]	Channel-by-epoch rejection retains more usable data	Robust feature extraction + artifact rejection
Fan et al. (2018) [328]	Group-level affect recognition feasible in VR driving	EEG-based affect recognition in VR
Fiebelkorn et al. (2013) [329]	Reduced category-based attention generalization	High-density EEG mapping
Fogelson et al. (2019) [330]	Attenuated P3b to predicted emotional faces	Predictive contextual processing EEG
Foss-Feig et al. (2018) [331]	Globally heightened P300 predicts psychosis conversion	P300 ERP analysis for psychosis prediction
Foss-Feig et al. (2018) [332]	Enhanced vs. attenuated P300 pattern differs by ASD	P300 amplitude analysis
Foss-Feig et al. (2021) [333]	Gap detection ERP correlated with symptom severity	Auditory gap detection ERP
Friedrich et al. (2015) [334]	Bidirectional mu training superior to unidirectional	Neurofeedback training effects
Frohlich et al. (2016) [335]	Higher beta and lower delta power in Dup15q	Beta/delta power biomarkers
Gabard-Durnam et al. (2013) [336]	Opposite alpha asymmetry trajectories by risk group	Frontal alpha asymmetry development
Gabard-Durnam et al. (2019) [337]	First-year EEG best discriminates ASD outcomes	Longitudinal EEG power analysis
Garces et al. (2022) [338]	No significant power/connectivity differences found	Resting state EEG power + connectivity
Ghanbari et al. (2015) [339]	Spatially complementary complexity–connectivity patterns	Joint complexity + connectivity analysis
Gialloreti et al. (2016) [340]	15% show improvements in both symptoms and skills	Longitudinal clinical evolution study
Glauser et al. (2022) [341]	P400 response correlated with future social skills	Face processing ERP analysis
Golob & Edgington (2016) [342]	Increased bottom-up attention correlated with sensitivity	Auditory ERP + sensory reactivity
Grossi et al. (2017) [343]	MS-ROM/I-FAST identifies brain disconnection patterns	MS-ROM/I-FAST + ML classification
Grujičić & Milovanovic (2021) [75]	U-shaped power pattern and E/I imbalance in ASD	EEG review paper
Gurau et al. (2017) [344]	Inconsistent results prevent diagnostic application	Systematic review of EEG utility
Haartsen et al. (2019) [345]	Fronto-central connectivity predicts restricted behaviors	EEG connectivity + behavioral correlation
Hadoush et al. (2019) [346]	Bilateral tDCS modulates resting EEG in ASD children	tDCS effects on resting EEG
Halliday et al. (2024) [347]	Substantial heterogeneity across all imaging modalities	Multi-modal neuroimaging review
Han et al. (2022) [348]	Multimodal fusion outperforms unimodal approaches	Multimodal EEG+eye-tracking + SDAE
Hasenstab et al. (2017) [349]	ASD shows different learning speed patterns	Multi-dimensional FPCA of ERP
Hasenstab et al. (2016) [350]	RFC algorithm accounts for ASD covariance heterogeneity	Robust functional clustering
Heunis et al. (2018) [351]	RQA promising but requires demographic control	Recurrence quantification analysis
Hu et al. (2023) [352]	Hierarchical regional-asymmetric features improve classification	Regional-asymmetric adaptive GCN
Huberty et al. (2021) [353]	High-risk infants show steeper power increase	Spectral power development analysis
Hudac et al. (2015) [354]	16p11.2 CNVs show opposite mu attenuation pattern	Mu attenuation to social stimuli
Isaev et al. (2020) [355]	RALD distinguishes ASD despite similar brain activity	Relative average look duration + EEG
Ja et al. (2008) [356]	All ASD children show perisylvian MEG abnormalities	MEG epileptiform activity analysis
Jaime et al. (2016) [357]	Reduced right temporal–central alpha coherence	Temporal–central alpha coherence
Jamal et al. (2014) [358]	Synchrostate network measures achieve high accuracy	Complex network measures + SVM
Jeste et al. (2015) [359]	Higher frontal theta correlated with poorer function	Resting EEG cognitive/language biomarkers
Jiang et al. (2022) [360]	EEG ideal for diagnosis, risk prediction, monitoring	EEG biomarkers review
Jones et al. (2017) [361]	Temporal variability changes predict rTMS efficacy	rTMS effects + temporal variability
Kala et al. (2021) [362]	Early intervention improves social attention measures	Parent-delivered intervention + EEG
Kang et al. (2020) [363]	N170 latency reduces after PRT intervention	N170 as treatment response biomarker
Kayarian et al. (2020) [364]	Combined EEG+eye-tracking achieves 85% accuracy	EEG+eye-tracking + SVM
Kirkovski et al. (2017) [365]	tES shows promise for enhancing gamma oscillations	Gamma pathophysiology review
Lauttia et al. (2019) [366]	Combined TMS-EEG reveals ASD neuropathophysiology	Combined TMS-EEG investigation
Li et al. (2024) [367]	ASD shows reversed pattern for direct vs. downcast gaze	Frontal EEG asymmetry to gaze
Li et al. (2022) [368]	Resting EEG shows minimal discrimination in adults	Resting EEG features + ML
Maharatna & Billeci (2021) [369]	Deep learning promising but challenges remain	EEG-based ASD identification review
Maharatna & Das (2021) [370]	Alpha/theta complexity, delta linearity as biomarkers	Linear+nonlinear IMF analysis
Mahmud & Wang (2024) [371]	Theta/alpha bands most discriminative for connectivity	PLV connectivity + graph theory + ML
Mash et al. (2018) [372]	ASD shows higher delay, more short-distance connections	Multivariate visibility graph model
Maxwell et al. (2015) [373]	Multimodal integration critical for resolving discrepancies	Multimodal connectivity review
McEvoy & Jeste (2014a) [374]	Reduced right lateral gamma correlated with severity	Resting gamma laterality analysis
McPartland et al. (2004) [375]	Frontal theta power associated with cognitive deficits	Resting EEG cognitive biomarkers
McPartland et al. (2014) [376]	Reduced P2 attention to social exclusion	Social exclusion ERP dynamics
McPartland et al. (2011) [377]	Psychophysiology has broad translational potential	Psychophysiological research review
McVoy et al. (2019) [378]	Decreased coherence consistent finding in ASD	qEEG biomarkers systematic review
Milne et al. (2009) [379]	Reduced alpha/gamma modulation in visual cortex	ICA visual perception analysis
Murias et al. (2018) [380]	Increased theta, decreased alpha coherence patterns	Resting EEG coherence analysis
Murias et al. (2007) [381]	Baseline beta power predicts treatment response	EEG biomarkers predict treatment response
Murphy et al. (2014) [382]	No task-dependent alpha modulation in ASD	Alpha-band suppression mechanisms
Naumann et al. (2018) [383]	Prolonged gamma elevation during holistic processing	Holistic face processing EEG
Neuhaus et al. (2021) [384]	Sex differences in frontal alpha correlations	Frontal alpha asymmetry by sex
Neuhaus et al. (2023) [385]	Decreased alpha power, sex-specific associations	Resting EEG by age/sex/phenotype
Nordt et al. (2016) [386]	Repetition suppression ideal for developmental studies	Repetition suppression paradigms
Nowicka et al. (2016) [387]	Absent self-preference effect, disrupted connectivity	Name recognition EEG + connectivity
Oliveira et al. (2018) [388]	E/I disbalance correlated with autistic trait severity	E/I disbalance as ASD predictor
Orekhova et al. (2014) [389]	Alpha hyper-connectivity predicts later ASD diagnosis	EEG hyper-connectivity in high-risk infants
Pandya et al. (2023) [390]	AI shows promise across multiple modalities	AI approaches in autism review
Parmar et al. (2021) [391]	HD-tDCS safe but no cognitive flexibility improvement	HD-tDCS cognitive flexibility study
Peck et al. (2021) [392]	Predictive features shift from 6 to 12 months	Language EEG prediction (6/12 months)
Peters et al. (2013) [393]	ASD shows decreased long/short-range connectivity ratio	Graph theory EEG connectivity
Petersons et al. (2021) [394]	Reduced NAA, increased rCBF, altered DTI metrics and metabolic dysfunction as converging biomarkers of ASD’s underlying neurophysiology	Multimodal MRI integration approach (combining DTI, ASL, and MR spectroscopy)
Piazza et al. (2023) [395]	ASD risk shows reduced low-frequency power	Baseline EEG spectral power analysis
Piccardi et al. (2021) [396]	Reduced neural repetition suppression predicts ASD traits	Tactile repetition suppression EEG
Pillai et al. (2018) [397]	ASD shows increased rather than decreased connectivity	Task-related connectivity modulation
Pineda et al. (2014) [398]	Neurofeedback normalizes behavior and electrophysiology	Neurofeedback training effects
Ranaut et al. (2024) [399]	EEG+ML integration enhances ASD identification precision	EEG+ML for ASD identification review
Righi et al. (2014) [400]	Reduced connectivity emerges by 12 months	First-year functional connectivity
Rogala et al. (2023) [401]	Combined statistical and ML approaches enhance reliability	Traditional stats + ML EEG analysis
Sahin & Sahin (2017) [402]	ERPs show translational potential as biomarkers	ERP circuit integrity assessment
Shou et al. (2017) [403]	Hyper-connectivity within, hypo-connectivity between ICNs	Intrinsic connectivity networks
Simon et al. (2017) [404]	Sensory hyporesponsiveness linked to increased connectivity	Sensory hyporesponsiveness EEG
Siper et al. (2018) [405]	VEPs examine E/I imbalance in Phelan–McDermid syndrome	VEP biomarker discovery
Siper et al. (2016) [406]	Reduced VEP amplitudes suggest altered E/I activity	Transient VEP assessment
Sotoodeh et al. (2018) [407]	Biological motion perception preserved in ASD	Biological motion perception EEG
Spiegel et al. (2019) [408]	Slower binocular rivalry predicts symptom severity	Binocular rivalry EEG dynamics
Sprengers et al. (2017) [409]	E/I ratio successfully classifies ASD subtypes	EEG-based decision support system
Stroganova et al. (2012) [410]	Different timing/topography of contour responses	High-frequency oscillatory response
Sundaresan et al. (2021) [411]	Two-layer LSTM optimal for anxiety classification	Two-layer LSTM RNN stress classification
Takarae et al. (2022) [412]	Microstate C frequency/duration atypical in ASD	EEG microstates analysis
Talebi et al. (2019) [413]	Higher linear connectivity in TD, nonlinear in ASD	nCREANN nonlinear connectivity
Tan et al. (2022) [414]	ET-EEG correlative analytics reveal developmental insights	Eye-tracking EEG correlative analytics
Tarasi et al. (2023) [415]	Predictive strategies track autism–schizophrenia continuum	Predictive strategies neural signatures
Tawhid et al. (2021) [416]	Deep learning outperforms traditional ML approaches	Spectrogram image + CNN classification
Tessier et al. (2015) [417]	Different sleep-dependent face processing networks	REM sleep + emotional face memory
Tierney et al. (2012) [418]	Dynamic developmental trajectories differ by risk group	Developmental EEG power trajectories
Torres et al. (2021) [419]	ROAR algorithm enables interpretable feature relevance	Interpretable deep learning evaluation
Tseng et al. (2024a) [420]	Lower LPP, larger P200, reduced theta synchronization	Game-based stimuli + mobile EEG
Tseng et al. (2024b) [421]	Reduced neural responses and functional connectivity	Game-based social interaction EEG
Uddin (2015) [422]	Brain state crucial for observing connectivity differences	Brain state functional connectivity
Valakh (2015) [423]	Homeostatic framework integrates contradictory E/I findings	E/I balance + circuit homeostasis
Van Hecke et al. (2015) [424]	PEERS intervention shifts to left-hemisphere dominance	PEERS intervention EEG asymmetry
Vettori et al. (2020) [425]	Reduced face saliency in superimposed stimulus streams	Frequency-tagging social stimuli
Vettori et al. (2019) [426]	Reduced social bias in both overt and covert measures	Combined frequency-tagging + eye tracking
Vilela et al. (2024) [427]	Genetic variants affect social cognition brain circuits	Genetic insights + neuroimaging review
Wang et al. (2013) [428]	U-shaped power profile, reduced long-range coherence	Resting state EEG abnormalities
Wilkinson et al. (2020) [429]	Early EEG highly predictive of 24-month language	Longitudinal EEG language development
Wright et al. (2012) [430]	Abnormal induced gamma to emotional faces	Gamma activation face emotion processing
Xu et al. (2024) [431]	Short-distance parietal/occipital differences predominant	Combined CNN-LSTM time series maps
Yardeni et al. (2021) [432]	Mitochondrial defects sufficient for ASD endophenotypes	Mitochondrial deficiency mouse model
Yeung et al. (2016) [433]	Reduced late-stage frontal theta in cognitive flexibility	Frontal theta oscillations analysis
Yu & Zhang (2019) [434]	Attenuated LRTC in social function brain regions	Long-range temporal correlations
Yu et al. (2024) [435]	Grid-tuned ensemble enhances classification stability	Grid-tuned ensemble 2D spectrogram
Zeng et al. (2017) [436]	Reduced whole-brain connectivity, disrupted organization	Disrupted brain network analysis
Zhang et al. (2022a) [437]	Graph-based dual-modal outperforms single-modal	Graph-based dual-modal features
Zhang et al. (2022b) [438]	EEG metrics effectively predict ADOS symptom severity	EEG metrics predict symptom severity

**Table 4:** Comprehensive AI algorithm classification and performance.

Algorithm Category	Specific Method	Studies (*n*)	Best Performance	Advantages	Limitations	Optimal Applications
Support Vector Machines	RBF Kernel	31	99.91% accuracy	High accuracy, interpretable	Limited to moderate datasets	Early detection, classification
	Linear SVM	12	87–92%	Fast training, simple	Limited nonlinear relationships	Feature selection
Deep Learning	3D-CNN	18	90–95%	Spatial relationships preserved	High computational cost	Volumetric data analysis
	CNN+SVM Hybrid	8	87.8%	Combined strengths	Complex architecture	EEG classification
	LSTM/RNN	10	83–89%	Temporal dynamics	Sequential data dependency	Longitudinal analysis
Ensemble Methods	Random Forest	24	85–92%	Robust, feature importance	Black box nature	Multi-feature datasets
	Gradient Boosting	8	88–93%	High accuracy	Overfitting risk	Complex feature spaces
	Stacking	6	90–95%	Leverages multiple models	High complexity	Multimodal integration
Traditional ML	Logistic Regression	15	75–85%	Interpretable, fast	Limited complexity handling	Baseline comparisons
	k-NN	8	70–82%	Simple, no training	Sensitive to noise	Small datasets
Specialized Methods	Graph Neural Networks	12	85–95%	Network topology	Complex implementation	Connectivity analysis
	Autoencoders	6	80–88%	Unsupervised learning	Requires large datasets	Feature learning

**Table 5:** Algorithm performance by data type and preprocessing methods.

Data Type	Preprocessing Method	Optimal Algorithm	Feature Extraction	Accuracy Range
Raw EEG Signals	DBSCAN Artifact Removal	SVM (RBF)	DFA, Entropy, Synchronization	90.57–99.91%
	ICA-AROMA	CNN	Wavelet Transform	85–92%
EEG Spectral Features	Bandpass Filtering	Random Forest	Power Spectrum, Coherence	80–95%
	CSP	k-NN + LBP	Spatial Patterns	85–90%
fMRI Time Series	Motion Correction + CompCor	Graph Theory + ML	Connectivity Matrices	75–88%
	ICA Denoising	CNN	Spatial-Temporal Features	82–90%
DTI Tractography	TBSS Preprocessing	SVM	FA, MD, Network Metrics	70–85%
Multimodal Data	Cross-Modal Registration	Fusion Algorithms	Combined Features	85–95%

## Data Availability

Not applicable.

## Supplementary Materials

The following supporting information can be downloaded at https://www.mdpi.com/article/10.3390/healthcare13151776/s1. Table S1. Full table of the studies (n = 146) included in the systematic review; Table S2. Comprehensive dataset reference table of the studies (n = 146); Table S3. Abbreviation list.
