# Supplementary material for: Leveraging AI-Driven Neuroimaging Biomarkers for Early Detection and Social Function Prediction in Autism Spectrum Disorders: A Systematic Review
Source: Healthcare (Basel). 2025 Jul 22;13(15):1776. doi: 10.3390/healthcare13151776 (PMC12346713; doi:10.3390/healthcare13151776)
Supplement: Supplementary file 1 [file healthcare-13-01776-s001.zip › Sup_Table_S3_Abbreviation List.pdf]

Table S3. Abbreviation List

Neuroimaging & Brain Imaging

AAL - Automated Anatomical Labeling
BOLD - Blood-Oxygen-Level-Dependent
CSD - Current Source Density
DTI - Diffusion Tensor Imaging
EEG - Electroencephalography
ERP - Event-Related Potential
FA - Fractional Anisotropy
fMRI - Functional Magnetic Resonance Imaging
fNIRS - Functional Near-Infrared Spectroscopy
HD-tDCS - High-Definition transcranial Direct Current Stimulation
MEG - Magnetoencephalography
MD - Mean Diffusivity
MRI - Magnetic Resonance Imaging
MRS - Magnetic Resonance Spectroscopy
NODDI - Neurite Orientation Dispersion and Density Imaging
PAF - Peak Alpha Frequency
qEEG - Quantitative EEG
REM - Rapid Eye Movement
ROI - Region of Interest
rsEEG - Resting-State EEG
rTMS - repetitive Transcranial Magnetic Stimulation
sMRI - Structural Magnetic Resonance Imaging
SNR - Signal-to-Noise Ratio
SSVEP - Steady-State Visual Evoked Potential
TBSS - Tract-Based Spatial Statistics
tDCS - transcranial Direct Current Stimulation
tES - transcranial Electrical Stimulation
TFD - Time-Frequency Distribution
TMS - Transcranial Magnetic Stimulation
VEF - Visual Evoked Field
VEP - Visual Evoked Potential

Artificial Intelligence & Machine Learning

ABIDE - Autism Brain Imaging Data Exchange
AI - Artificial Intelligence
AUC - Area Under the Curve
BCI - Brain-Computer Interface
CCA - Canonical Correlation Analysis
CNN - Convolutional Neural Network
CSP - Common Spatial Pattern
DBSCAN - Density-Based Spatial Clustering of Applications with Noise
DFA - Detrended Fluctuation Analysis
DL - Deep Learning
FPCA - Functional Principal Component Analysis
GAN - Generative Adversarial Network
GNN - Graph Neural Network
ICA - Independent Component Analysis
ITT - Intention-to-Treat
k-NN - k-Nearest Neighbor
LASSO - Least Absolute Shrinkage and Selection Operator
LBP - Local Binary Pattern
LDA - Linear Discriminant Analysis
LIME - Local Interpretable Model-agnostic Explanations
LR - Logistic Regression
LSTM - Long Short-Term Memory
ML - Machine Learning

mMSE - Modified Multiscale Entropy
MSE - Multiscale Entropy
MVPA - Multivariate Pattern Analysis
PCA - Principal Component Analysis
PLV - Phase Locking Value
RF - Random Forest
RFE - Recursive Feature Elimination
RNN - Recurrent Neural Network
ROC - Receiver Operating Characteristic
RQA - Recurrence Quantification Analysis
SHAP - Shapley Additive exPlanations
SVM - Support Vector Machine
VAE - Variational Autoencoder
WPLI - Weighted Phase Lag Index
XGBoost - eXtreme Gradient Boosting

Clinical & Behavioral Assessment

ADI-R - Autism Diagnostic Interview-Revised
ADHD - Attention-Deficit/Hyperactivity Disorder
ADOS - Autism Diagnostic Observation Schedule
ASD - Autism Spectrum Disorder
CARS - Childhood Autism Rating Scale
DSM - Diagnostic and Statistical Manual
HRA - High Risk for Autism
HR-ASD - High Risk with ASD diagnosis
HR-NoASD - High Risk without ASD diagnosis
ICD - International Classification of Diseases
LRC - Low-Risk Control
M-CHAT - Modified Checklist for Autism in Toddlers
PEERS - Program for the Education and Enrichment of Relational Skills
PRT - Pivotal Response Treatment
SCQ - Social Communication Questionnaire
SRS - Social Responsiveness Scale
TD - Typically Developing
ToM - Theory of Mind
TSC - Tuberous Sclerosis Complex
VABS - Vineland Adaptive Behavior Scales

Neuroscience & Brain Function

CEN - Central Executive Network
CNV - Copy Number Variant
DMN - Default Mode Network
dlPFC - Dorsolateral Prefrontal Cortex
E/I - Excitation/Inhibition
FPN - Frontoparietal Network
GABA - Gamma-Aminobutyric Acid
ICN - Intrinsic Connectivity Network
LPP - Late Positive Potential
LRTC - Long-Range Temporal Correlations
mmN - Mismatch Negativity
mPFC - Medial Prefrontal Cortex
N170 - N170 component (ERP)
P100 - P100 component (ERP)
P200 - P200 component (ERP)
P300 - P300 component (ERP)
P400 - P400 component (ERP)
pSTS - posterior Superior Temporal Sulcus
RewP - Reward Positivity
SN - Salience Network
SNP - Single Nucleotide Polymorphism

SPN - Stimulus Preceding Negativity
STS - Superior Temporal Sulcus
TPJ - Temporoparietal Junction

Research Methodology & Standards

ANCOVA - Analysis of Covariance
ANOVA - Analysis of Variance
ComBat - Combating Batch Effects
CompCor - Component-based Noise Correction
CPAC - Configurable Pipeline for the Analysis of Connectomes
FDR - False Discovery Rate
FWE - Family-Wise Error
FWER - Family-Wise Error Rate
GLM - General Linear Model
HAPPE - Harvard Automated Preprocessing Pipeline for EEG
ICA-AROMA - ICA-based Automatic Removal of Motion Artifacts
ICC - Intraclass Correlation Coefficient
JBI - Joanna Briggs Institute
NIAK - NeuroImaging Analysis Kit
OSF - Open Science Framework
PRISMA - Preferred Reporting Items for Systematic Reviews and Meta-Analyses
PROSPERO - International Prospective Register of Systematic Reviews
RETROICOR - RETROspective Image CORrection
RoB - Risk of Bias
TFCE - Threshold-Free Cluster Enhancement

Organizations & Datasets

CDC - Centers for Disease Control and Prevention
EMA - European Medicines Agency
EU-AIMS - European Autism Interventions
FDA - Food and Drug Administration
IBIS - Infant Brain Imaging Study
LEAP - Longitudinal European Autism Project
NIH - National Institutes of Health
NIMH - National Institute of Mental Health
WHO - World Health Organization

Medical & Clinical Terms

AE - Adverse Event
BBB - Blood-Brain Barrier
CNS - Central Nervous System
CSF - Cerebrospinal Fluid
GCP - Good Clinical Practice
IRB - Institutional Review Board
SAE - Serious Adverse Event

Technology & Computing

API - Application Programming Interface
CPU - Central Processing Unit
CSV - Comma-Separated Values
GPU - Graphics Processing Unit
Hz - Hertz
IDE - Integrated Development Environment
JSON - JavaScript Object Notation
kHz - Kilohertz
NoSQL - Not Only SQL
RAM - Random Access Memory
SDK - Software Development Kit
SQL - Structured Query Language
XML - eXtensible Markup Language
